# Horse Clinical Cytogenetics: Recurrent Themes and Novel Findings

**DOI:** 10.3390/ani11030831

**Published:** 2021-03-16

**Authors:** Monika Bugno-Poniewierska, Terje Raudsepp

**Affiliations:** 1 Department of Animal Reproduction, Anatomy and Genomics, University of Agriculture in Krakow, 31-120 Krakow, Poland; 2 Department of Veterinary Integrative Biosciences, Texas A&M University, College Station, TX 77843-4458, USA

**Keywords:** horse, chromosome aberration, aneuploidy, translocation, structural rearrangements, sex reversal, chimerism, molecular cytogenetics, FISH, CGH

## Abstract

**Simple Summary:**

Horse chromosomes have been studied for veterinary diagnostic purposes for over half a century. The findings show that changes in the chromosome number or structure are among the most common non-infectious causes of decreased fertility, infertility, and developmental abnormalities. Based on large-scale surveys, almost 30% of horses with reproductive or developmental problems have abnormal chromosomes. For a comparison, only 2–5% of horses in the general population have abnormal chromosomes. Most chromosome abnormalities are rare and found in one or a few animals. However, two conditions are recurrent: sterile mares with only one X chromosome, instead of two, and sterile mares with XY male sex chromosomes where the Y has lost the ‘maleness’ gene *SRY.* The two are signature features of chromosome abnormalities in the horse, being rare or absent in other domestic animals. The progress in horse genome sequencing and the development of molecular tools have improved the depth and quality of diagnostic chromosome analysis, allowing for an understanding of the underlying molecular mechanisms. Nevertheless, cutting-edge genomics tools are not about to entirely replace traditional chromosome analysis, which still is the most straightforward, cost-effective, and fastest approach for the initial evaluation of potential breeding animals and horses with reproductive or developmental disorders.

**Abstract:**

Clinical cytogenetic studies in horses have been ongoing for over half a century and clearly demonstrate that chromosomal disorders are among the most common non-infectious causes of decreased fertility, infertility, and congenital defects. Large-scale cytogenetic surveys show that almost 30% of horses with reproductive or developmental problems have chromosome aberrations, whereas abnormal karyotypes are found in only 2–5% of the general population. Among the many chromosome abnormalities reported in the horse, most are unique or rare. However, all surveys agree that there are two recurrent conditions: X-monosomy and *SRY*-negative XY male-to-female sex reversal, making up approximately 35% and 11% of all chromosome abnormalities, respectively. The two are signature conditions for the horse and rare or absent in other domestic species. The progress in equine genomics and the development of molecular tools, have qualitatively improved clinical cytogenetics today, allowing for refined characterization of aberrations and understanding the underlying molecular mechanisms. While cutting-edge genomics tools promise further improvements in chromosome analysis, they will not entirely replace traditional cytogenetics, which still is the most straightforward, cost-effective, and fastest approach for the initial evaluation of potential breeding animals and horses with reproductive or developmental disorders.

## 1. Introduction

Clinical cytogenetic research in horses has been ongoing for over half a century and has clearly demonstrated that chromosome abnormalities are associated with congenital disorders, embryonic loss, reduced fertility, and infertility. Changes in the chromosome number or structure typically result in genomic imbalance and affect meiotic cell division, gametogenesis, and the viability of zygotes and embryos. Genetically balanced chromosomal changes, such as translocations, can be transmitted, causing fertility problems in subsequent generations. In cases where chromosomal aberrations do not show phenotypic or behavioral effects, the carriers can be included in breeding, resulting in significant economic loss due to veterinary fees and the costs related to maintaining a sterile or a subfertile horse over the years. Therefore, the cytogenetic screening of potential breeding animals and clinical cytogenetic evaluation of problem horses are of economic importance for the equine industry, as well as for the owners and breeders.

During the peak of equine clinical cytogenetics in the 1970s–1990s, many abnormal karyotypes were published and in the following years, and the findings have been well-reviewed in books [1,2], book chapters [3,4,5,6,7], and multiple review papers, some specifically focusing on equine cytogenetics [8,9,10], others on cytogenetics of domestic animals, including the horse [11,12,13,14]. Since the last comprehensive and horse-focused reviews about a decade ago [4,5], equine clinical cytogenetics has advanced qualitatively, mainly thanks to the progress in equine genomics and the availability of new powerful genomic tools (reviewed by [15]). At the same time, the quantity of clinical cytogenetic publications in the horse has reduced compared to the pinnacle times in 1970s–1990s. This, however, is not because there are less horses with karyotype aberrations but rather because not every cytogenetic case results in a publication. The majority of recent reports combine conventional cytogenetics with molecular methods which allow for the validation and refinement of the findings, but also start revealing the underlying molecular causes and mechanisms of chromosome abnormalities in the horse.

In this review, we appraise the cytogenetic findings of the past and combine those with recent reports to identify novel findings and highlight recurrent patterns of chromosome abnormalities in the horse. We also discuss how molecular tools and the availability of the horse reference genome have essentially advanced equine clinical cytogenetics today and what the perspectives for the future are.

## 2. The Horse Chromosomes

### 2.1. Chromosome Number

The first reports about horse chromosomes date back to the early 20th century, when, using spermatogonial and meiotic preparations, it was proposed that the horse diploid number is approximately 20–37 [16,17,18] with an XO sex chromosome system [19]. Thanks to improvements in the chromosome analysis methodology, these early findings were soon revised showing that like in other mammals, horse has XY sex chromosome system [20] and the correct diploid number for the domestic horse (*Equus caballus*, ECA) is 2n = 64 [21,22,23].

### 2.2. Application of Different Chromosome Banding Techniques

Horse cytogenetics has evolved in conjunction with human cytogenetics and adopted from the latter all main chromosome differential staining or banding techniques (reviewed by [3]). Of the many techniques developed in the 1970s, only a few have remained in active everyday use in equine clinical chromosome analysis. Among these, the most common are G-banding [24] and its fluorescent version with 4′,6-diamidino-2-phenylindole, known as DAPI-banding [25]. The latter produces G-band-like pattern and is an essential part of all molecular cytogenetic methods (see Section 4). C-banding [26] is an excellent method to visualize horse sex chromosomes and is still in use as an additional method in cases involving sex chromosome abnormalities [27,28]. Compared to these, R-banding [29] and NOR-banding [30] are predominantly used for research [31,32,33] and not for routine clinical karyotyping.

### 2.3. Karyotype Features and Chromosome Nomenclature

In order to properly characterize chromosome abnormalities and communicate the findings between cytogenetic laboratories, standard karyotypes, and chromosome nomenclatures have been developed. These are agreements among researchers worldwide about how to number the chromosomes and arrange them by size, centromere position, and specific banding patterns, and demarcate individual chromosomal regions and bands.

To date, three standard karyotypes have been developed for the horse, the first from 1980 [34] and the second from 1990 [35], mainly differed by chromosome arrangement and numbering, but provided detailed description of the horse karyotype and chromosome banding patterns and established common grounds for clinical cytogeneticists. The third and current standard karyotype from 1997 [36] maintained the second arrangement [35] but included an enumerated nomenclature of chromosome bands.

According to ISCNH 1997 [36], the autosomes are divided into two groups: in the first group there are 13 pairs of meta- and sub-metacentric chromosomes, the second group includes 18 pairs of acrocentric chromosomes. Within each group, the autosomes are ordered by length. The sex chromosomes—a sub-metacentric X chromosome and an acrocentric Y chromosome [37]—are located in the center of the karyogram, next to the row of the three smallest bi-armed chromosomes. Horse sex chromosomes show distinct C-banding patterns. The X chromosome has two C-bands, one corresponding to the pericentromeric heterochromatin, another to an ampliconic array of *ETSTY7* sequences [38] interstitially in Xq17. The *ETSTY7* sequences prevail in the Y chromosome, which is almost completely C-band positive. Heterochromatin-rich pericentromeric C-bands are also present in most of the autosomes, except chr11. The latter is devoid of centromeric satellite DNA and presents an example of a chromosome with a neo-centromere where centromere function precedes satellite repeat accumulation [39]. The standard also describes the location of the 18S-5.8S-28S ribosomal RNA gene clusters or nucleolus organizer regions (NORs), which are in the telomeric region of chr1 and in the secondary constriction in chr28 and chr31. Some studies also found NOR in chr27 [40,41], though a more recent study [33] did not confirm the presence of a fourth pair of an NOR-bearing chromosome.

## 3. Chromosome Aberrations

### 3.1. Emerging Patterns of Chromosome Abnormalities—Large-Scale Studies

Diagnostic cytogenetic research in horses dates back to the late 1960s, preceding the introduction of chromosome banding techniques [42]. The first karyotype abnormalities detected by banding methods were published by Chandley et al. in 1975 [43]: in 7 mares referred for research due to reproductive problems, aneuploidies 63,X and 63,XXX; mosaicism 63,X/64,XX and 64,XY sex reversal were identified. Over the following years, several large-scale cytogenetic surveys [8,44,45,46] started to reveal the most prevalent and specific patterns of chromosome abnormalities in the horse.

A study of 180 mares with gonadal dysgenesis [44] found chromosomal abnormalities in 54%. The most common abnormality was X-monosomy (63,X), followed by 64,XY male-to-female sex reversal syndrome. Two mares showed structural abnormalities of one X chromosome [64,X,del(Xp)]. Chromosomal abnormalities, such as 63,X; 63,X/64,XX; 64,X,del(Xp) and 64,XX,i(26q), were also found in 4 fillies that were tested due to their small size and poor thriving [44].

A survey by Power [8], recorded X-monosomy in 204 mares (51%) out of 401 tested horses with chromosomal abnormalities. Of these, 70% had non-mosaic X-monosomy. Like in the survey by Bowling et al. [44], the second most frequent karyotype aberration was XY sex reversal, which was diagnosed in 27% out of the 401 horses. Over 13% of horses had various non-mosaic and mosaic forms of sex chromosome aneuploidies, such as 65,XXX; 65,XXY; 66,XXXY; 64,XX/65XXX; 63,X/64XY; 63,X/65,XYY; 64,XX/65,XXY; 63,X/64,XX/64,XY; 63,X/64,XY/65,XXY; 63,X/64,XX/65,XXY; 64,XX/64,XY/65,XXY; 63,X/64,XX/64,XY/65,XXY and 63,X/64,XX/65,XXX/65,XXY/66,XXXY/66,XXYY). The remaining 6% had structural aberrations (translocations, deletions, isochromosomes) or autosomal trisomies.

A third survey by Parada et al. [45] examined 244 mares with reproductive problems. Chromosome aberrations were found in 10 of the studied mares, which accounted for 4% of the entire study population, and 12.8% of completely sterile mares [45]. Like in the two previous large-scale surveys [8,44], the most common aberration was X-monosomy with non-mosaic 63,X in 3 mares, and mosaic 63,X/64,XX in four mares, which in total accounted for 70% of all aberrations [45]. Other findings included XX/XY leukocyte chimerism and an elongation of the p-arm of chr 12 [45]. The prevalence of sex chromosome abnormalities was also reported by a smaller-scale cytogenetic analysis of 42 mares with reproductive problems [47,48] showing mosaicism 63,X/64,XX in five individuals and a three-cell line mosaicism 63,X/64,XX/65,XXX in one mare. In addition, the analysis of two fillies and one colt from two different-sex twin pregnancies revealed one pair of twins with lymphocyte chimerism 64,XX/64,XY.

In order to find out the prevalence of chromosome abnormalities in general horse populations, Bugno et al. [46] conducted cytogenetic analysis in 500 young horses—272 fillies and 228 colts of 10 diverse breeds and breed crosses. Karyotype abnormalities were found in 10 young mares, which accounted for 2% of the entire population and 3.7% of the female population [46]. Among the diagnosed aberrations, 8 were X chromosome aneuploidies (80%)—one pure 63,X and seven 63,X/64,XX mosaics, one case of XX/XY chimerism, and one case of mosaicism for trisomy 31 (64,XX/65,XX,+31).

The above described surveys of reproductively abnormal and general horse populations were all conducted using conventional cytogenetic techniques. However, the development of molecular cytogenetic methods (see Section 4 for details), has increased the accuracy and power of diagnosis. For example, two recent studies validated the results of prior cytogenetic findings in a population of 500 young (up to 2 years) horses using fluorescence in situ hybridization (FISH) with molecular probes specific for the horse sex chromosomes [49,50]. The preliminary karyotyping results of 238 horses showed normal female or male karyotype in 225 animals. In 13 horses (5.5%) the following aberrations were found: 63,X/64,XX (3 mares); 63,X/64,XX/65,XXX (1 mare); 64,XX/65,XXX (2 mares); 64,XX/64,XX,del(Xp) (1 mare); 63,X/64,XX del(X)?/64,XX (1 mare); 63,X/64,XX/65,XXX del(X)? (1 mare); 64,XY/65XYY (1 stallion); 64,XX/64,XY (1 stallion); 64,XY *SRY*-negative sex reversal (1 mare), and one mare with a reciprocal translocation between chromosome 1 and X: 64,X,t(1p;Xp)(1q;Xq).

**Figure 1:**
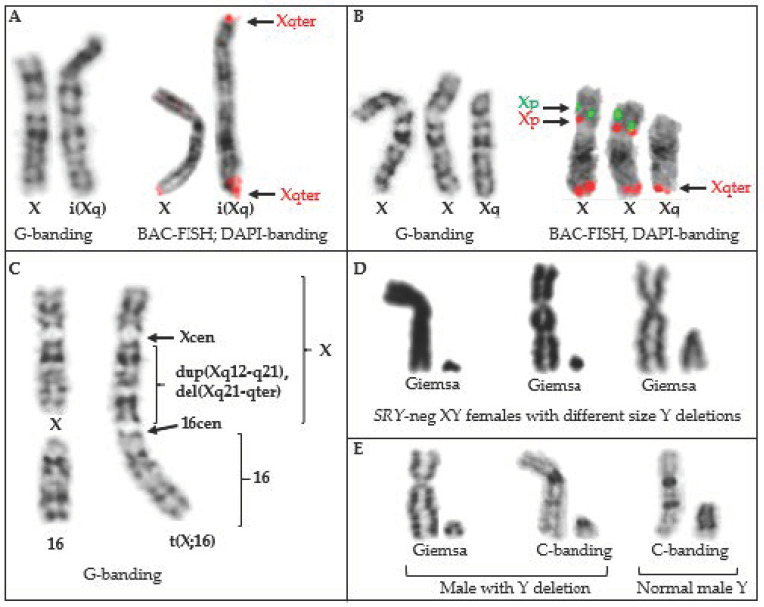
Examples of horse sex chromosome structural rearrangements (TAMUMCL archive). (**A**) Isochromosome Xq in a mare with short stature, no ovaries and 64,X,i(Xq) karyotype; (**B**) Trisomy Xq in a non-cycling mare with 65,XX,+Xq karyotype; (**C**) Complex dicentric X-autosome rearrangement in a mare with short stature but no other obvious problems, fertility unknown; (**D**) Sex chromosomes of three mares with *SRY*-neg XY male-to-female sex reversal syndrome; the first two have large deletions in the Y, the third one has a cytogenetically normal-looking Y, but a submicroscopic deletion around the *SRY* gene; (**E**) Partial Y chromosome deletion in a Shetland pony without penis (left, middle), comparison with the sex chromosomes of a normal male (right).

Finally, over the past 20 years (2001–2021), the Texas A&M Molecular Cytogenetics Laboratory (TAMUMCL) has analyzed 766 horses with congenital abnormalities, disorders of sex development, and/or reproductive problems, using a combination of conventional and molecular cytogenetic approaches. The data (Table 1, Figure 1) show that 28% of problem horses have karyotype abnormalities and like in all previous large-scale surveys, the most prevalent chromosome abnormalities are X-monosomy (35% of all chromosome abnormalities; 10% of all problem horses; 18% of all problem females) and *SRY*-negative XY sex reversal (11% of all chromosome abnormalities; 3% of all problem horses; 6% of problem females).

### 3.2. Sex Chromosome Aneuploidies

As shown by large-scale surveys and by many individual case reports (reviewed by [3,5,9,10,51]), the most common karyotype abnormality in horses worldwide is X-monosomy (63,X) and its mosaic forms 63,X/64,XX and 63,X/64,XY. Occasionally, X-monosomy has been found together with a second, also abnormal cell line, e.g., 63,X/65,XXX [59] or 63,X/65,XYY [60,61], or as a mosaic of several cell lines [62,63,64,65,66,67].

***X-monosomy.*** Mares with X-monosomy are often characterized by a lower height than age- and breed-mates with normal karyotype. They usually have properly developed external genitalia but have often underdeveloped small hypoplastic ovaries with no palpable follicles, and a small and flaccid uterus. Mares with X-monosomy show decreased steroidogenic activity of the ovaries and have overall higher levels of the luteinizing hormone, and lower levels of estrogen, progesterone, testosterone and cortisol [45,68,69]. The consequence of these changes are disturbances in the development and functioning of the reproductive system, leading to the absence of the estrus cycle and sterility. While the typical consequence of non-mosaic X-monosomy is sterility, a few cases of foals born to mares with a mosaic karyotype 63,X/64,XX have been described [8,44,47,66,69,70,71].

***Sex chromosome trisomies—XXX, XXY and XYY.*** The second type of aneuploidy diagnosed in horses is sex chromosome trisomy—the presence of supernumerary X or Y chromosomes. These abnormalities are rare and like X-monosomy occur as non-mosaic 65,XXX [43,72,73,74,75] and 65,XXY [76,77,78], or as a mosaic of two [59,79,80,81], or more cell lines [46,63,82,83,84]. Mares with X-trisomy may look phenotypically normal. Some, especially those with mosaic X-trisomy, may show signs of estrus, but are rarely able to produce offspring because of hypoplastic gonads [59,74,85]. Likewise, stallions with XXY sex chromosomes may look normal and show normal male behavior but are sterile due to testicular hypoplasia and azoospermia [76,77,78,79,86]. Cases of male horses with an extra Y chromosome (65,XYY) are rare, show various forms of Disorders of Sex Development (DSDs), and have been described as pseudohermaphrodites [60,61,64]. In the 1970s, it was believed that the presence of two Y chromosomes could positively affect the performance of stallions. However, the cytogenetic research carried out among champions at that time did not confirm these expectations [87].

### 3.3. Autosomal Aneuploidies

Autosomal aneuploidies are rare in horses because the resulting genetic imbalance is typically lethal, and the few reported live-born cases are exclusively trisomies (Table 2). Among the large-scale cytogenetic surveys discussed in Section 3.1, autosomal aneuploidies were recorded only by two—the survey by M. Power [8] and TAMUMCL 20-year data. In the latter, autosomal trisomies were found in just 4 animals out of the 766 abnormal horses studied, and account for less than 2% of all detected chromosome abnormalities (Table 1). In contrast, a recent whole genome analysis identified autosomal aneuploidies (both trisomies and monosomies) in over 20% of equine early pregnancy losses (EPLs) at 14–65 days of gestation [88]. This is in line with the data for humans where autosomal aneuploidies are well understood and described, and account for approximately 50% of all diagnosed chromosome disorders in miscarried fetuses [89,90].

To date, 14 liveborn cases with trisomies involving 6 autosomes have been reported and in all, the extra chromosome is one of the smallest acrocentrics (Table 2). Phenotypes of the carriers vary but typically have numerous severe congenital malformations and primary infertility. The first diagnosed case was trisomy 28 in a Thoroughbred male with very short stature, cryptorchidism and azoospermia [91]. A foal with trisomy 23 had numerous defects of the skeletal system and sexual organs [84]. Trisomy 26 has been reported twice: in a filly with poor constitution, neurologic and behavioral issues ([92], and a colt with neurologic and gait defects and poor thriving (TAMUMCL). Curiously, in both cases, the chromosome number was normal 2n = 64, either due to the formation of isochromosome 26 or by Robertsonian fusion of the extra chr26. The dams of the abnormal foals in both cases were relatively young (3 years-old and 5 years-old, respectively), thus excluding advanced maternal age as a contributing factor. However, the most notable is that the filly with trisomy 26 turned into a fertile mare who gave birth to a healthy and chromosomally normal colt [92]. Trisomy 27 has been found in 4 cases and in all, the affected foals had multiple congenital malformations, including contracted tendon [93], arthrogryposis [94], skeletal malformations [95], and gait and behavioral abnormalities [57]. Four cases have also been diagnosed with trisomy 30, all having multiple developmental and behavioral abnormalities, such as poor thriving (TAMUMCL cases); abnormal gait and limb malformations [92]; facial deformities, scoliosis and heart and artery defects [95]. In contrast, trisomy for the smallest equine autosome, chr31, has been reported only once—a colt with underdevelopment of the limbs and reproductive organs [96].

Primary infertility, which is associated with the majority of non-mosaic autosomal trisomies, prevents propagating the aberrations in the population. However, this is not the case of mosaic forms. For example, Kubień and Tischner [97] described a phenotypically normal Polish Konik mare with 64, XX/65,XX 0 karyotype that gave birth to a normal foal. Likewise, Bugno et al. [46] diagnosed a 64,XX/65,XX,+31 karyotype in a few months-old filly with no developmental anomalies at this age. The lack of developmental abnormalities and normal fertility in mosaic forms of autosomal trisomy is likely due to the presence of a cell line with a normal karyotype.

It is well-established that the risk for autosomal trisomies in humans increases with advancing maternal age [98]. No such statistics is available for the horse, mainly because of the small number of reported cases. However, in some of the above-described studies, advanced age of the dam has been considered as a contributing factor [44,92]. In others, however, foals with an autosomal trisomy have been born to mares of average reproductive age (Table 2). Likewise, no clear correlation between maternal age and aneuploidies were detected in the single study of EPLs [88]. Because horses are used for breeding at all ages, continuing collection of cases with phenotypic and parental information is needed to shed more light into this matter.

### 3.4. Structural Rearrangements

Structural rearrangements change the constitution of one or more chromosomes and are typically caused by double-stranded DNA breaks and subsequent mistakes in repair during meiosis [99]. Depending on their effect on genome integrity, structural rearrangements are classified as genetically balanced and unbalanced. Balanced rearrangements include inversions and most translocations, and do not change the DNA content of a cell. Balanced structural changes typically do not have noticeable phenotypic manifestation and can easily remain unnoticed in carrier animals. In contrast, unbalanced rearrangements, such as deletions, duplications, and unbalanced translocations, cause a gain or loss of the genetic material and depending on the size and content, may have more or less severe effects on development, viability and reproduction (reviewed by [13,14]).

***Translocations.*** Translocations involve nonhomologous chromosomes which exchange parts or fuse, giving rise to reciprocal or nonreciprocal translocations, respectively [100]. Carriers of genetically balanced translocations appear phenotypically normal but have reduced fertility because of producing both genetically balanced and unbalanced gametes. The former can pass the translocation between generations, whereas fertilization of unbalanced gametes typically results in embryonic or fetal death, and is noticed as reduced fertility [14,54]. Carriers of unbalanced translocations, on the other hand, show a range of developmental and reproductive disorders depending on the extent of genetic imbalance and the regions involved [100].

Translocations are rare in horses and to date, only 15 unique translocations have been reported (Table 3). Of these, 11 are autosomal and 4 involve an autosome and a sex chromosome.

*Autosomal translocations.* The majority of autosomal translocations found in horses are balanced, thus not affecting the performance or appearance of the carrier animal. They were discovered because the carrier mare or stallion was subjected for chromosome analysis due to recurrent early embryonic loss (REEL) and subfertility (reviewed by [4,5,9,14]). Therefore, the actual frequency of balanced autosomal translocations in equine populations may be higher, but due to no phenotypic effect and because only select individuals are used for breeding, they remain undetected [54]. In contrast, the single case of a live horse with unbalanced autosomal translocation, a Warmblood colt with 64,XY,t(4;30),+4p (Table 3), was euthanized due to poor thriving [54]. Overall, live animals with unbalanced autosomal translocations are extremely rare and typically, the condition is not viable beyond preimplantation [101].

Balanced translocations are among the few hereditary chromosome abnormalities because the carriers can pass the condition to their offspring. If transmitted, the translocation will cause similar subfertility issues in the next generation [14]. In horse breeding where sires and dams are selected based on their athletic performance, appearance, and pedigrees, rather than reproductive performance, this can lead to propagating translocations over generations. Currently, there is cytogenetic evidence for three such ’translocation families’ (Table 3): an elite Thoroughbred stallion with 64,XY,t(5;16),+mar passing the rearrangement to 8 offspring [4]; an elite Warmblood stallion with 64,XY,t(4;30),der(4q) passing the rearrangement to 5 offspring [54], and a Thoroughbred mare with 64,XX,t(2;13) passing the rearrangement to a single foal [54,56]. Therefore, systematic chromosome analysis of prospective breeding animals is needed for early detection of translocation carriers to prevent transmission and reduce economic loss due to subfertility.

*Autosome and sex chromosome translocations.* The phenotypic effects of translocations involving sex chromosomes differ from those of autosomes, as well as from each other. In each case, the genetic consequences depend on whether the horse is male or female and which X chromosome regions are involved. This is because random X inactivation (XCI) in mammalian females [106,107], balances X chromosome gene dosage between sexes, but also buffers deleterious effects of X chromosome mutations [108,109]. This is illustrated by phenotypic differences between the three reported cases of X-autosome translocations (Table 3). The mare with a balanced 64,X,t(1p;Xp)(1q;Xq) karyotype was phenotypically normal [49]. The Thoroughbred mare with unbalanced, dicentric complex X chromosome rearrangement and t(16;X) (Figure 1C), had only mild phenotype with short stature. This is because there was no autosomal imbalance and the rearranged X chromosome portion was subject for XCI, which probably did not spread over to chr16 [27]. In contrast, the Thoroughbred mare with unbalanced 64,X,t(15;X),-Xp,+15 karyotype had a short stature and was infertile [91], which is consistent with monosomy for Xp [14]. However, this mare also had trisomy 15, which most likely should not be viable, but because one copy of chr15 was translocated to Xq, it was functionally silenced by XCI [91].

A unique case is a balanced reciprocal Y-chr13 translocation in a Friesian stallion with complete azoospermia [52]. It is the first case of azoospermia in stallions with a cytogenetically detected Y chromosome abnormality. However, because balanced translocations typically cause only subfertility, the complete meiotic arrest and azoospermia in this stallion remained a puzzle [52]. The mystery was resolved by a recent hypothesis about Y-linked meiotic executioner genes which are necessary for successful meiosis but must also be subjected to meiotic sex chromosome inactivation (MSCI) [110]. If such genes are translocated to an autosome, ectopic expression of these genes during MSCI results in fatal meiotic arrest [110]. Thus, the Friesian stallion with Y-autosome translocation is a proof-of-principle to this hypothesis and another example illustrating different genetic consequences of translocations involving autosomes only compared to those involving an autosome and a sex chromosome.

*Translocation-prone chromosomes.* Even though none of equine translocations have been recurrent, i.e., have not independently occurred in unrelated individuals, some horse chromosomes tend to be engaged more often than others. For example, only 14 autosomes (out of 31) and the sex chromosomes have been involved in the 15 currently known translocations (Table 3) [54]. Of these, chr1 has been involved five times, chr16 four times, chr4, 13, and X three times each, chr30 twice, and chr2, 3, 5, 10, 12, 17, 21, 22, 25, and Y once each. Whether or not the involvement of particular chromosomes is random or associated with specific molecular features, remains a topic for future research. Studies in humans and pigs, where translocation frequency is high, indicate that translocation breakpoints are not random and occur preferentially in regions with open chromatin (G-negative bands), higher gene density and common fragile sites, and are demarcated by repetitive elements such as LINEs, SINEs, and endogenous retroviral elements [99,111]. Continuing the collection of additional clinical and cytogenetic data on translocations is the key for revealing their molecular patterns in horse chromosomes.

***Deletions and duplications.*** Deletions and duplications decrease or increase the total amount of DNA in a cell, respectively, and cause genomic imbalance. Loss or gain of large chromosomal segments is usually lethal or accompanied by severe malformations and infertility. Smaller submicroscopic deletions and duplications, also known as DNA copy number variants (CNVs), may or may not have any evident phenotypic effect, and their contribution to equine health and fertility is, as of yet, poorly understood [112].

Chromosomal deletions and duplications are part of unbalanced translocations and were discussed in the previous section (Table 3). Otherwise, there are just two reports on cytogenetically detected autosomal deletions: deletion of chr13qter [64,XY,del(13)(qter)] in a Standardbred stallion [63] and an Arabian stallion with mosaicism for XX/XY and chr10 deletion [64,XY/63,XY,–10; 64,XX/63,XX,–10;] [113]. Both cases were identified due to fertility issues. However, it must be noted that these studies predated the availability of molecular cytogenetic tools to validate the findings.

Horse Y chromosome is particularly prone for deletions, most of which cause *SRY*-negative XY sex reversal syndrome and are discussed in Section 3.6 and Section 5. In addition, TAMUMCL has studied a case of a male Shetland pony with no penis. The horse was *SRY*-positive and had 64,XY karyotype with an unusually small Y chromosome that had lost the majority of the C-band positive heterochromatin (Table 1, Figure 1E).

***Inversions.*** An inversion occurs when a piece of a chromosome breaks and reinserts within the same chromosome in inverted orientation [100]. Inversions are hard to detect both by conventional and molecular cytogenetic approaches, and to date there are no reports about equine clinical cases caused by inversions. The only known cytogenetically detectable inversion in the horse is the over 40 Mb-size inversion in chr3q causing the tobiano color pattern but is not associated with a disease or disorder [114].

***Isochromosomes.*** Isochromosomes (i) are structurally abnormal chromosomes that are formed through a centric mis-division and result in chromosome arms which are mirror image of each other and genetically identical [115]. If one copy of the normal chromosome is also retained, the result is trisomy for that chromosome arm. Thus, isochromosome formation is both a structural and numerical rearrangement.

**Figure 2:**
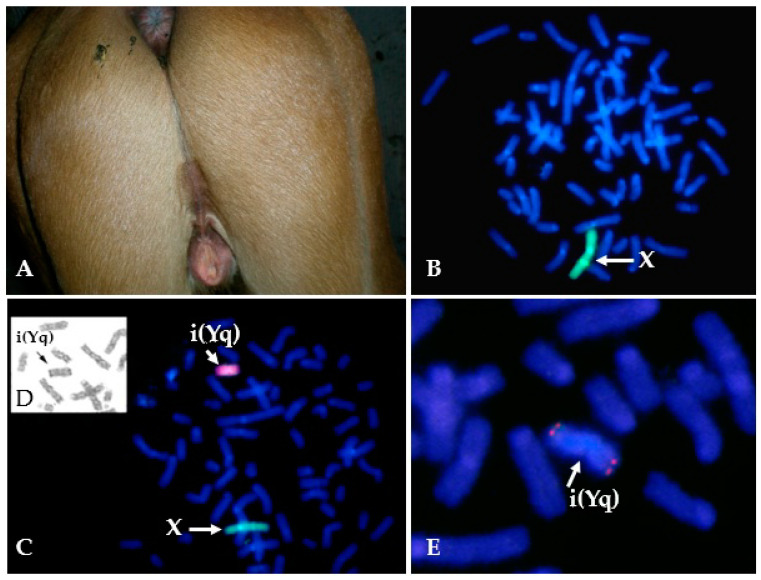
Example of a horse with isochromosome Y in a mosaic karyotype 63,X/64,X,i(Yq). (**A**) Abnormal external genitalia of a 6 month-old mare; (**B**) Cell line with X-monosomy 63,X (97%); the single X is labeled green with X-specific painting probe; (**C**) Cell line with isochromosome Y and 64,X,i(Yq) karyotype (3%); X is labeled green with X-specific painting probe and Y is labeled red with Y-specific painting probe; (**D**) Part of the same metaphase after G-banding showing i(Yq) (arrow); (**E**) Partial metaphase after FISH with a probe specific for *USP9Y* region in Y (note the red signal at both ends of the isochromosome Y).

Isochromosomes have been reported for horse sex chromosomes –i(Xq) ([116]; TAMUMCL, Table 1, Figure 1A) and i(Yq) [37,117,118]. The two cases of 64,X,i(Xq) ([116]; TAMUMCL) were both described as having short stature and small inactive ovaries—a typical phenotype for X-monosomy or the deletion of Xp [14]. Isochromosome Y has been found only in mosaic form as 63,X/64,X,i(Yq) [37,117,118] (Figure 2). Two cases had similar DSD phenotypes—a male pseudohermaphrodite [117] and an intersex [37]. The third case was slightly different describing a female horse with abnormal external genitalia (Figure 2), but here the researchers also detected by PCR analysis Y chromosome microdeletions [118].

Among autosomes, there are two cases with putative isochromosome 26—a fertile Thoroughbred mare [92] and a Thoroughbred colt with neurologic issues, gait problems and poor growth (TAMUMCL, Table 1). Both horses had normal chromosome number with 64,XX or 64,XY karyotypes, respectively, but carried one normal chr26 and one metacentric marker chromosome which both arms corresponded to chr26. Further analysis by microsatellite genotyping (see Section 4.3) is needed to reveal whether the abnormal metacentric chromosome is an isochromosome or a result of Robertsonian fusion. In the first case, all chr26 markers should be bi-allelic, in the latter, three alleles can be detected. Regardless, recurrence of i(26q) or rob(26q26q) in unrelated horses is certainly a curious observation.

***Fragile sites.*** Fragile sites are specific chromosomal loci that exhibit gaps and breaks on metaphase chromosomes following partial inhibition of DNA synthesis. Common fragile sites are found in all individuals, while rare fragile sites occur infrequently, are inherited in the Mendelian manner and can be associated with congenital disorders [119,120].

Chromosomal fragility has also been reported in horses in connection with sterility and reduced fertility [32], though the mechanism underlying this connection remains unclear [119]. Difficulty of interpretation is probably one of the reasons why fragile sites or chromosome breaks have not been included in any equine clinical cytogenetic case report, even though breaks and gaps have been observed and recorded in laboratory notes (TAMUMCL archive). Fragile sites certainly deserve further attention by clinical cytogenetics and basic genome research because in both humans and horses, they have been co-localized with interstitial telomeric sequences, known as genomic ‘scars’ marking DNA break/repair sites and possibly more unstable genomic regions [121].

### 3.5. Chimerism

The term *chimera* is defined as an individual that has two cell lines derived from two separate zygotes. This disorder may appear as a result of the early fusion of zygotes, which are the result of fertilization of the egg and polar body. In a situation where their genetic sex is different (XX and XY), chimerism involving all tissues leads to intersex with ovotestis. Chimerism may also be caused by early embryo fusion [122].

The best-known form of chimerism in mammals is the presence of two cell populations in individuals born from twin or multiple pregnancy, both the same-sex and different-sex. In these cases, the occurrence of cellular chimerism is the result of the formation of a common bloodstream through anastomoses, i.e., vascular connections between the fetal membranes of co-twins [123,124]. The consequence is the exchange of hematopoietic cells and the interaction of the endocrine and immune systems between the twins. The consequence of the formation of anastomoses are changes in the female reproductive system, which most often lead to infertility (cattle, sheep) or reproductive problems (horses) [53,125,126,127,128,129,130,131].

The cases of fertile mares with XX/XY leukocyte chimerism may indicate a late contact of placental vessels between fetuses [129]. If the fusion takes place after sex determination and differentiation towards ovaries, they do not undergo masculinization, which occurs in freemartin heifers derived from different-sex twin pregnancies [131].

### 3.6. Cytogenetics of Sex Reversal Conditions

The term *sex reversal* has been used to describe situations where the genetic sex as determined by sex chromosomes does not agree with the gonadal and/or external phenotypic sex. In human medicine, due to social and ethical issues, gender-based diagnostic labels such as *sex reversal, intersex*, *hermaphroditism*, and *pseudohermaphroditism*, have been replaced by a more neutral term *disorders of sex development* (*DSDs*) [132]. The term has also been extended to veterinary medicine to denote congenital conditions in which development of chromosomal, gonadal, or anatomic sex is atypical [11]. Conditions with a normal karyotype but atypical or ambiguous sex development are classified according to the sex chromosomes into XY and XX DSDs.

***64,XY DSDs.*** Many cases of male-to-female sex reversal or XY DSDs have been described in horses and after X-monosomy, they are the most common equine chromosome abnormalities (reviewed by [4,5,10,11,12,13,14,133,134]). It has been estimated that approximately 12% to 30% of all cytogenetically abnormal cases are XY DSDs [28,44,135] (Table 1).

In earlier studies, when XY DSDs were recognized solely by karyotyping, without testing for the *SRY* gene, the researchers observed that phenotypes of XY sex reversal horses can vary in a broad range—from a very feminine to a greatly masculinized mare [44,135,136,137]. With the inception of *SRY* testing in equine cytogenetics, first by Southern blotting [138] and thereafter by PCR [73,139,140], horse XY DSDs are categorized as *SRY*-negative and *SRY*-positive.

*SRY-negative XY DSDs.* This is the most prevalent form of XY DSDs and encompasses the majority of the feminine-type cases. The affected mares typically have normal female external genitalia and no somatic or behavioral abnormalities but are sterile due to ovarian and uterine dysgenesis [10,11,28,73,78,133,138,141,142,143,144]. The phenotype of *SRY*-negative XY DSD very closely resembles that of X-monosomy, indicating that while the absence of *SRY* blocks the male development pathway in these individuals, normal female development still requires the presence of two X chromosomes. At molecular level, *SRY*-negative XY DSD in horses is caused by Y chromosome deletions (Figure 1D) and discussed in detail in Section 6.

*SRY-positive XY DSDs.* This group of XY DSDs encompasses female-like horses showing various degrees of masculinization and virilization, as well as stallion-like behavior. These horses usually have abnormally developed genital tract, the gonads can range from ovotestes to testicular feminization, and the cases are described as male pseudohermaphrodites, intersex or ambiguous sex [28,44,133,134,136,137,145,146,147,148,149].

Despite female-like appearance, these horses are genetically male with an intact Y chromosome and a normal *SRY* gene [28]. Molecular causes for abnormal sex development are known or suggested only for a few cases. In three families of different breeds, *SRY*-positive XY DSD was associated with androgen insensitivity syndrome and with different mutations in the androgen receptor gene [150,151,152]. In two related male pseudohermaphrodite Standardbreds with *SRY*-positive XY DSD [146], a large (~200 kb) homozygous deletion in chr29 was found and proposed as a likely cause because the deletion removed a cluster of genes (*AKR1C* family) with known functions in steroid hormone biosynthesis, including androgens and estrogens [58,112]. However, *AR* mutations or the deletion in chr29 are not present in many other cases of *SRY*-positive XY DSDs [58], suggesting that the molecular causes of the condition are heterogeneous.

***64,XX DSDs.*** Horses with XX DSDs are *SRY*-negative and cytogenetically indistinguishable from normal females. However, all equine XX DSDs cases have highly abnormal and ambiguous sex phenotypes (reviewed by [10,12,13,14]). In contrast to humans where multiple cases of *SRY*-positive XX males have been reported [153], true XX female-to-male sex reversal condition has not been found in horses. Over the years, tens of XX DSD cases have been described [133,139,154,155,156,157,158,159,160], including 41 unpublished cases from TAMUMCL (Table 1). While clinical details of individual cases may vary, they are typically reported as intersex, hermaphrodite or ambiguous sex because of difficulties to decide about the gonadal and/or phenotypic sex of the horse (reviewed by [134]). Molecular causes of equine XX DSD are unknown.

## 4. Molecular Cytogenetic Methods and Applications

During the past three decades, largely thanks to the progress in horse gene mapping and genome sequencing (reviewed by [15]), methodological advancements have also shaped equine cytogenetics, leading to improved resolution and accuracy in detecting various types of chromosome abnormalities. Clinical cytogenetics today is essentially a combination of conventional chromosome analysis by banding techniques and a variety of molecular approaches.

### 4.1. Fluorescence In Situ Hybridization (FISH)

The most widely used molecular approach in equine clinical cytogenetics is fluorescence in situ hybridization (FISH). The method was developed in the 1980s (reviewed by [161]), relies on the Watson–Crick DNA base-pairing complementarity principle and permits identification of the location of DNA sequences in their original place (in situ) in mitotic and meiotic chromosomes at different stages of the cell cycle [162,163]. The most commonly used probes for FISH in cytogenetic studies are clones from the horse genomic bacterial artificial chromosome (BAC) library CHORI-241 (https://bacpacresources.org/ accessed on 1 March 2021). This is because thanks to the whole-genome radiation hybrid and FISH map [164] and available end sequence data for approximately 315,000 BACs [165], precise chromosomal and sequence map locations are known for thousands of clones from this library. Therefore, if a BAC clone is needed for FISH to identify the chromosomes involved in numerical or structural rearrangements or for determining rearrangement breakpoints, it can be found from the Genomic Clones track of EquCab3 assembly in NCBI Genome (https://www.ncbi.nlm.nih.gov/genome/?term=horse accessed on 1 March 2021) and ordered from the CHORI BACPAC resources (https://bacpacresources.org/ accessed on 1 March 2021).

Other important FISH probes are horse chromosome-specific paints generated by chromosome flow sorting [72,166] or microdissection [167,168,169,170]. The latter method also allows for the preparation of probes specific for chromosomal segments and has been used to generate painting probes for the short- and long arm of the horse X chromosome [171].

In addition to BACs and chromosome painting probes, FISH probes are available for vertebrate telomeric repeats (Discovery^®^: https://www.discoverypeptides.com/pna/pna-telomere-fish-probes accessed on 1 March 2021), multicopy 18S-5.8S-28S ribosomal DNA (rDNA) sequences [33], also known as nucleolus organizer regions (NORs), and horse centromeres [172]. Alternatively, researchers have used *primed* in situ *DNA* synthesis (PRINS) for the detection of telomere, centromere, and rDNA repeat sequences in horse chromosomes [33,173,174].

### 4.2. Application of FISH in Horse Clinical Cytogenetics

The first application of FISH in equine clinical cytogenetics was the use of a flow sorted X chromosome paint to detect X chromosome aneuploidies [72]. Since then, X chromosome paints have been used in multiple cytogenetic cases for the detection of mosaic (see Figure 2B,C) and non-mosaic X chromosome aneuploidies [64,74,167,169], aneuploidies of X chromosome arms [171], sex chromosome mosaicism [175], and in one recent case, to show premature X chromosome centromere division in a Hucul mare [176]. Combination of both the X and the Y chromosome paints (Figure 2C) or the Y paint alone, have been used to confirm sex chromosome complement and large Y chromosome deletions in cases of XY male-to-female sex reversal [28,141].

In contrast to the wide use of sex chromosome paints, there are no reports about FISH with horse autosomal paints to analyze cases of autosomal aneuploidies or structural rearrangements. So far, all FISH experiments validating and refining various horse translocations (Table 3) have used BAC clones. Likewise, BAC-FISH has also been instrumental for the accurate identification of the small autosomes involved in trisomies [57,95] (Table 2), for confirming isochromosome formation [37], and for characterizing two cases with complex structural rearrangements. The first one involved 5;16 translocation and a de novo small marker chromosome [4], another had a dicentric X;16 translocation with partial Xq duplication and deletion [27]. The use of BACs instead of chromosome paints in these cases is probably because BAC-FISH provides better resolution for resolving rearrangement breakpoints, but also because horse chromosome painting probes are not commercially available.

Compared to BACs and sex chromosome paints, the use of FISH with centromeric, telomeric, or rDNA probes in equine clinical cytogenetics has been limited. The few examples include centromere-FISH to locate centromeric sequences in a dicentric derivative chromosome [27] and show the position of centromeres in isochromosome Y [37]. The latter study also determined that the horse Y chromosome is an acrocentric and not sub-metacentric as presented in ISCNH 1997 [36].

### 4.3. Cytogenetic Evaluation of Stallions by Sperm-FISH

Sperm-FISH is a state-of-art technique to analyze chromosomal constitution of mature spermatozoa, which have highly condensed chromatin, do not undergo cell division and cannot be studied by conventional cytogenetic approaches. Sperm-FISH is carried out on decondensed sperm nuclei using chromosome-specific paints or BAC clones. The method was initially developed for men [177] but has been optimized for domestic species, including the stallion [178]. While karyotyping evaluates chromosomes in diploid somatic cells, sperm-FISH allows determining the chromosomal constitution of mature haploid sperm and is potentially more informative for fertility evaluation. However, the ability to detect aneuploidies is limited to the availability of chromosome-specific probes and the number of fluorochromes that can be simultaneously visualized under the microscope.

Owing to these limitations, sperm-FISH studies in stallions have been restricted to analyzing sex chromosome aneuploidies in reproductively normal [179,180,181] and subfertile stallions [182]. These studies indicate that sex chromosome aneuploidy rate in normal stallions is in the range of 0.32–1.14% [179,180,181] with the highest frequency for sex chromosome nullisomy (0.47–1.22%) and the lowest for trisomy XXX or XXY (0.008–0.02%) [180,181]. Correlation has also been found between stallion age and the total number of aberrations in sperm [180,181]. Compared to normal stallions, subfertile Sorraia stallions have over five times more (5.83%) sex chromosome aneuploidies [182], whereas stallions in general show the highest rate of X and Y aneuploidies among domestic species (reviewed by [14]). Based on these data, it is tempting to speculate that the relatively high frequency of X-monosomy found in horses (see Section 3.2.) is partially caused by sperm aneuploidies, particularly the sex chromosome nullisomy.

### 4.4. Whole Genome Analysis by Comparative Genomic Hybridization and Sequencing

Comparative genomic hybridization (CGH) was originally designed for human cancer cytogenetics to overcome the difficulties to obtain high-quality metaphase spreads from various solid tumors [183]. The technique uses competitive hybridization of two differently labeled (red and green) DNA probes, one from a normal control, another from a cancer cell to normal metaphase chromosomes. The measurement of the ratios of red-to-green fluorescence along chromosomes will identify gains and losses in the cancer genome compared to the control. In horses, the CGH technique has been used to identify chromosome rearrangements involving large deletions and amplifications in equine sarcoid cells [184]. With the development of array technology, CGH has been adapted for SNP and oligonucleotide tiling arrays, known as array CGH (aCGH) [185,186].

The contribution of aCGH to horse clinical cytogenetics has been limited to just four studies. The first one used the Equine SNP50 BeadChip (Illumina) to confirm previously known cases of X monosomy and trisomy 31 and identified new cases with trisomy 27 and 31 [95]. The second study, used aCGH to investigate CNVs in normal horse genome, but coincidentally discovered a large, over 200 kb deletion in chr29 of two female-like horses with 64,XY *SRY*-positive DSD [112]. The third study applied aCGH to identify chromosome rearrangements in an intersex horse [187]. The most recent study used the high density 670K equine SNP array [188] and detected 12 different, mostly novel, autosomal aneuploidies in fetuses from early pregnancy loss [88] (Table 2). The findings were confirmed by whole genome sequencing (WGS) and digital droplet PCR, suggesting that advanced molecular methods will gradually become an integral part of equine clinical cytogenetics.

### 4.5. Immunolocalization of Chromosomal Proteins

The use of fluorescently labeled antibodies for chromosomal proteins such as centromere kinetochore proteins, synaptonemal complex proteins SCP1 and SCP3, proteins associated with double stranded break repair and recombination or meiotic silencing, has considerably improved the knowledge about chromosome function in normal cells, as well as in cells with chromosomal aberrations (reviewed by [189]). Immunostaining is often combined with FISH, which further increases the power and resolution of analysis.

In horse cytogenetics, immunostaining has been used to understand the organization, function, and evolution of centromeres [190,191] and for the study of synaptonemal complexes, recombination sites and the chiasmata in meiosis prophase and MI of normal stallions [192,193]. To date, immunostaining has not been used for the study of aberrant horse chromosomes.

### 4.6. STR Genotyping in Cytogenetics—Advantages and Limitations

Short tandem repeats (STR), also known as microsatellites, are widely used markers in parentage testing. The International Society of Animal Genetics (ISAG) recommends the use of a properly standardized panel of 17 microsatellite markers for horses, located on 12 different autosomes and the X chromosome. By adding to this set additional X and Y chromosome markers, whole-genome STR genotyping for parentage testing can simultaneously be used for the initial detection of chromosomal aberrations, such as monosomy, trisomy, XY sex reversal syndrome and chimerism [53,126,127,194,195,196]. Microsatellite genotyping has also been used to determine the parental origin of an aberrant chromosome [27,197] and in a study of two cloned horses, one with a de novo autosomal translocation, to confirm that the clones and their sire were genetically identical [54]. Other potential uses of STR genotyping are the identification of isochromosomes and determining the parental origin of the single X chromosome in X-monosomy. The latter will improve our currently limited understanding about the underlying mechanisms of this most common cytogenetic abnormality in horses.

The greatest advantages of this method are high sensitivity and specificity, speed of analysis, ease of interpretation of the results, and relatively low cost. As discussed in Section 3, chromosomal aberrations are most often associated with disorders of reproductive function, which are the most common reason for referral of horses for cytogenetic diagnostic testing. Therefore, in many cases, chromosome abnormalities are detected in adult individuals. Parentage tests, on the other hand, are usually performed in yearlings, which allows for the early diagnosis of any problems. It should be emphasized that DNA for STR analysis can be isolated from tissues other than blood and does not require lymphocyte culture for several days. Despite the many advantages, STR genotyping also has limitations in karyotype analysis. It cannot detect balanced structural aberrationsor aneuploidy in a mosaic form Though, in cases of X-monosomy, STR genotyping can be used to exclude mosaicism.

## 5. Molecular Underpinnings of the Unique Patterns of Horse Chromosome Abnormalities

All large-scale cytogenetic surveys (see Section 3.1) unanimously agree that the two most frequent chromosome abnormalities in the horse are X-monosomy and *SRY*-negative XY male-to-female sex reversal syndrome (*SRY*-negative XY DSD). The high prevalence of the two conditions is a signature feature of equine clinical cytogenetics, with no similar patterns found in other domestic species [14,28,51]. Recent advances in horse genomics (reviewed by [15]), particularly in the genomics of horse sex chromosomes [38,198], start to provide the first clues for these signatures.

***X-monosomy.*** The high frequency of viable X-monosomy in horses, but not in other domestic species, has been associated with the molecular features of the horse pseudoautosomal region (PAR) [51]. The equine PAR is approximately 2 Mb in size [199], which is several magnitudes smaller than the 6–9 Mb-size PARs in other domestic species, such as cattle, sheep, goat, pig, camelids, dog and cat [14,51,198]. Since PAR genes escape X chromosome inactivation (XCI) in females and must be expressed bi-allelically, X-monosomy causes haploinsufficiency for these genes. It has been theorized that because the horse PAR is relatively small, less genes are affected by X-monosomy, resulting in viable live birth, whereas X-monosomy in species with larger PARs causes embryonic or fetal loss [14,51]. An alternative hypothesis, however, proposes that the rate of sex chromosome rearrangements, including aneuploidies, increases when the PAR shrinks because reduced X-Y synapsis in male meiosis causes more mistakes [200]. Both theories are consistent with the relatively high frequency of X-monosomy in humans (0.04% of live female births) [201], which is another species with a small PAR (2.7 Mb) [202]. However, the small PAR does not explain the dramatic differences between horses and humans regardings X chromosome and PAR overdose. Compared to X-monosomy, XXX or XXY aneuploidies are rare in the horse and the few reported cases show gonadal dysgenesis and infertility (see Section 3.2). In contrast, the XXY Klinefelter’s syndrome and X-trisomy are the most common sex chromosome abnormalities in humans, affecting 0.1–0.2% of male births [203] and 0.1% of female births [204], respectively. Furthermore, many women with X-trisomy are fertile, thus potentially increasing the number of XXY male births. It has been proposed that the low number of identified 65,XXX horses is because the majority of mares with X-trisomy are normal fertile and escape detection [8]. However, in such cases, the incidence of 65,XXY male horses should be higher. A more plausible explanation is that despite similar PARs, the molecular regulation of the X chromosome in horses and humans is different, though more research is needed to confirm this.

***SRY-negative 64,XY sex reversal.*** Thanks to recent sequencing of the horse Y chromosome [38], more is known about the molecular underpinnings of the relatively high frequency of mares with *SRY*-negative XY DSD. It appears that the single-copy horse *SRY* is located in a structurally unstable region in the Y chromosome, being embedded between ampliconic sequences and surrounded by direct and inverted repeats [38]. Such a location facilitates *SRY* involvement in ectopic inter-and intra-chromatid gene conversion and recombination within the Y chromosome [205]. These events may result in *SRY* deletion in one sperm and duplication in another [28,38]. Therefore, *SRY*-negative XY DSD females may have male siblings with two copies of *SRY*. The latter probably has no effect on the phenotype and remains undetected. Since the organization and content of mammalian Y chromosomes is different across species [38], this also explains why *SRY*-negative XY sex reversal is rare or absent in other species studied, including humans. For example, only 10–20% of human XY females (Swyer syndrome) have *SRY* mutations and the majority carry normal *SRY* [206].

## 6. Summary and Future Directions

Equine clinical cytogenetics has come a long and eventful way since the first description of karyotype abnormalities in horses in 1975 [43]. Starting with basic karyotyping of routinely Giemsa-stained chromosomes, it soon developed into an international, actively publishing, and methodologically sophisticated field of research to study the genetic causes of equine reproductive and congenital disorders. Despite the predictions that with the development of molecular methods, classical chromosome analysis will gradually disappear, horse clinical cytogenetics has remained. It successfully survived the golden days of equine gene mapping in the 1990s by adopting molecular cytogenetic methods, such as FISH and CGH. It remained active during the years of horse genome sequencing by applying genomic information to make chromosome analysis more refined and accurate. In the post-genome era today, cytogenetic research is blending with whole genome sequencing (WGS) and other cutting-edge technologies. Among the latter, perhaps the most promising for clinical cytogenetics is BioNano Genomics, which utilizes nanochannel technology and high-resolution imaging of ultra-high molecular weight DNA. The platform can detect almost any structural or numerical changes in the genome, including balanced translocations and inversions [207], and differently from WGS, the estimated price per sample of BioNano analysis is comparable to that of conventional clinical cytogenetics. The downsides, however, are that BioNano sets extremely high requirements for sample quality, and both WGS and BioNano are bioinformatically demanding. Therefore, despite the promises of new technologies, it is unlikely that they will entirely replace conventional and FISH-based chromosome analysis. Traditional clinical cytogenetics is still the most straightforward, cost-effective, and fastest approach to diagnose chromosome abnormalities, and will remain the gold standard for the initial evaluation of potential breeding animals and horses with reproductive or developmental disorders.

## Figures and Tables

**Table 1:** Summary of clinical cytogenetic findings of the Texas A&M Molecular Cytogenetics Laboratory (TAMUMCL) in the period of 2001–2021.

Problem Horses	Total Number of Individuals	% of All Horses Studied	% of All Chromosome Abnormalities	Reference
Subjected for karyotyping due to reproductive or developmental problems	766	-	-	
Males	244	31.9	-	
Females	427	55.7	-	
Ambiguous sex	95	12.4	-	
Horses with chromosome abnormalities	215	28.1	-	
**Types of chromosome abnormalities**				
X-monosomy	76	9.9; (17.8) *	35.3	[51]
X-trisomy	5	0.7; (1.2) *	2.3	[51]
Sex chromosome and ploidy mosaicism 63,X/64,XY 64,XX/128,XXXX 63,X/64,X,i(Yq) (Figure 1A)	3	0.4	1.4	[37]
X chromosome structural rearrangements 64,X,del(Xp) 64,X,i(Xq); 2 cases 65,XX,+Xq (Figure 1B)	5	0.7	2.3	
X-autosome complex rearrangement 63,X,der(X),del(q22),dup(q21q11),t(X;16)(q21;q11),dic(X;16) (Figure 1C)	1	0.1	0.5	[27]
Y chromosome structural rearrangements 64,XY,del(Y)(q11q13) (Figure 1E)	1	0.1	0.5	
Y-autosome structural rearrangement 64,XY,t(Yq;13p)	1	0.1	0.5	[52]
XX/XY blood chimerism	4	0.5	1.9	[53]
Autosomal translocations 64,XX,t(2;13); familial, 2 cases 64,XX;t(4;10) 64,X?;t(5;16),+mar; familial, 9 cases 64,XY;t(12q;25q),der(12p) 64,XY;t(4p;30q); familial, 6 cases	20	2.6	9.3	[4,54,55,56]
Autosomal aneuploidies 65,XY+27 65,XY+30; 2 cases 64,XY,i(26q) or 64,XY,rob(26q26q)	4	0.5	1.9	[57]
XY Sex reversal conditions *SRY*-neg XY DSD females; 24 cases (Figure 1D) *SRY*-pos XY DSDs female-like; 20 cases	44 24 20	5.7; (10.3) * 3.1; (5.6) * 2.6; (4.7) *	20.5 11.2 9.3	[28,58]
XX DSDs; ambiguous sex	41	5.4	19.1	[58]
*SRY*-pos XY DSDs; male-like	8	1.0	3.7	[58]

* numbers in parentheses show percent of all problem females; DSD—Disorder of Sex Development.

**Table 2:** Summary data about all individual cases of autosomal aneuploidies reported for the horse.

Chr.	Karyotype/Type of Aneuploidy	Mosaicism	Phenotype	Methods	Breed	Maternal Age	Reference
1	n/a; trisomy	n/a	Early pregnancy loss fetus	SNP-CGH; WGS; ddPCR	WB	4	[88]
3	n/a; trisomy	n/a	Early pregnancy loss fetus	SNP-CGH; WGS; ddPCR	TB	6	[88]
15	n/a; trisomy	n/a	Early pregnancy loss fetus	SNP-CGH; WGS; ddPCR	TB	19	[88]
20	n/a; trisomy	n/a	Early pregnancy loss fetus	SNP-CGH; WGS; ddPCR	TB	13	[88]
20	n/a; trisomy	n/a	Early pregnancy loss fetus	SNP-CGH; WGS; ddPCR	TB	19	[88]
23	65,XY,+23	non-mosaic	Liveborn, congenital defects	G- and C-banding	STB	n/a	[84]
23, 24	n/a; double trisomy	n/a	Early pregnancy loss fetus	SNP-CGH; WGS; ddPCR	TB	3	[88]
26	64,XX,i(26q) or 64,XX,rob(26q26q)	non-mosaic	Liveborn, congenital defects, fertile	G-, R- and C-banding	TB	3	[92]
26	64,XX,i(26q) or 64,XX,rob(26q26q)	non-mosaic	Liveborn, congenital defects	G-banding; BAC-FISH	TB	5	TAMUMCL
27	65,XY,+27	non-mosaic	Liveborn, congenital defects	G-banding	QH	26	[93]
27	65,XY,+27	non-mosaic	Liveborn, congenital defects	G-banding	AR	25	[94]
27	65,XY,+27	non-mosaic	Liveborn, congenital defects	G-banding; BAC-FISH	STB	5	[57]
27	64,XX/65,XX,+27	mosaic	Liveborn, congenital defects	G-banding, BAC-FISH; SNP-CGH	FR	n/a	[95]
27	n/a; monosomy	n/a	Early pregnancy loss fetus	SNP-CGH; WGS; ddPCR	TB	10	[88]
27	n/a; monosomy	n/a	Early pregnancy loss fetus	SNP-CGH; WGS; ddPCR	TB	19	[88]
28	65,XY,+28	non-mosaic	Liveborn, congenital defects	G- and R-banding	TB	14	[91]
30	65,XX,+30	non-mosaic	Liveborn, congenital defects	G-, R- and C-banding	AR	23	[92]
30	n/a; trisomy	non-mosaic	Liveborn, congenital defects	SNP-CGH	WP	n/a	[95]
30	65,XX,+30	non-mosaic	Liveborn, congenital defects	G-banding; BAC-FISH	M	23	TAMUMCL
30	65,XY,+30	non-mosaic	Liveborn, congenital defects	G-banding; BAC-FISH	AR	9	TAMUMCL
30	64,XX/65,XX,+30	mosaic	Liveborn, fertile	G-banding	PK	n/a	[97]
30	n/a; trisomy	n/a	Early pregnancy loss fetus	SNP-CGH; WGS; ddPCR	TB	9	[88]
30	n/a; trisomy	n/a	Early pregnancy loss fetus	SNP-CGH; WGS; ddPCR	TB	19	[88]
31	65,XY,+31	non-mosaic	Liveborn, congenital defects	G-banding	TB	26	[96]
31	n/a; trisomy	n/a	Early pregnancy loss fetus	SNP-CGH; WGS; ddPCR	WB	10	[88]
31	64,XX/65,XX,+31	mosaic	Liveborn, normal	G-and C-banding	TB	n/a	[46]

CGH—comparative genomic hybridization; WGS—whole genome sequencing; ddPCR—digital droplet PCR; abbreviations of horse breeds: AR- Arabian; FR—Friesian; M—Morgan; PK—Polish Konik; QH—American Quarter Horse; STB—Standardbred; TB—Thoroughbred; WB—Warmblood; WP—Welsh Pony.

**Table 3:** Summary data of all translocations found in horses.

Karyotype	Type	Genetic Balance	Evidence of Transmission	Reproductive Phenotype	Methods	Breed	Reference
64,XX,t(1q;3q)	Reciprocal	balanced	no	REEL	G- and R-banding	TB	[102]
64,XY,t(1;30)	Tandem	balanced	no	subfertility	G- and C-banding	TB	[103]
64,XX,t(1;16)	Reciprocal	balanced	no	subfertility	G-and C-banding; BAC-FISH; Zoo-FISH	TB	[104]
64,XX,t(1;21)	nonreciprocal	balanced	no	REEL	G-and C-banding; BAC-FISH	TB	[105]
64,XX,t(2;13)	nonreciprocal	balanced	yes	REEL	G-banding; BAC-FISH	TB	[56]
64,XX,t(4;13)	Reciprocal	balanced	no	REEL	G-and C-banding; BAC-FISH	TB	[105]
64,XX,t(4;10)	nonreciprocal	balanced	no	REEL	G-banding; BAC-FISH	AR	[55]
64,XY,t(4;30),der(4q) and 64,XY,t(4;30),+4p	nonreciprocal	balanced/ unbalanced	yes	foals with congenital abnormalities	G-banding; BAC-FISH	WB	[58]
64,XY,t(5;16),+mar	nonreciprocal	balanced	yes	REEL	G-banding; BAC-FISH	TB	[4]
64,XY,t(12;25),der(12p)	nonreciprocal	balanced	no	azoospermia, small testes	G-banding; BAC-FISH	AR	[58]
64,XX,t(16;22),+mar	Reciprocal	balanced	no	REEL	G-and C-banding; BAC-FISH	TB	[105]
64,X,t(1p;Xp)(1q;Xq)	Reciprocal	balanced	no	n/a	G-banding; BAC-FISH	n/a	[49]
63,X,t(Xq;16),+ complex X rearrangements	nonreciprocal	unbalanced	no	n/a	G-banding; BAC-FISH	TB	[27]
64,X,t(15;X),-Xp,+15 *	nonreciprocal	unbalanced	no	infertility	G- and R-banding	TB	[91]
64,X,t(13;Y)	Reciprocal	balanced	no	azoospermia	G-and C-banding; BAC-FISH	FR	[52]

* According to ISCNH1997, the involved autosome is chr17; REEL—Recurrent Early Embryonic Loss; Abbreviations of horse breeds: AR—Arabian; FR-Friesian; TB—Thoroughbred; WB—Warmblood.

## Data Availability

Not applicable.

## References

[1] Bowling A.T. (1996). Horse Genetics.

[2] Bailey E., Brooks S.A. (2013). Horse Genetics.

[3] Chowdhary B.P., Raudsepp T., Bowling A.T., Ruvinsky A. (2000). Cytogenetics and physical gene maps. The Genetics of the Horse.

[4] Durkin K., Raudsepp T., Chowdhary B., Vaala W.E., Varner D.D. (2011). Cytogenetic Evaluation of the Stallion. Equine Reproduction.

[5] Lear T.L., Villagomez D.A.F., Vaala W.E., Varner D.D. (2011). Cytogenetic evaluation of mares and foals. Equine Reproduction.

[6] Penedo M.C.T., Raudsepp T., Chowdhary B.P. (2013). Molecular Genetic Testing and Karyotyping in the Horse. Equine Genomics.

[7] Raudsepp T., Das P.J., Chowdhary B.P. (2013). Genomics of reproduction and fertility. Equine Genomics.

[8] Power M.M. (1990). Chromosomes of the horse. Adv. Vet. Sci. Comp. Med..

[9] Lear T.L., Bailey E. (2008). Equine clinical cytogenetics: The past and future. Cytogenet. Genome Res..

[10] Lear T.L., McGee R.B. (2012). Disorders of sexual development in the domestic horse, *Equus caballus*. Sex. Dev..

[11] Villagomez D.A., Iannuzzi L., King W.A. (2012). Disorders of sex development in domestic animals. Preface. Sex. Dev..

[12] Villagomez D.A., Parma P., Radi O., di Meo G., Pinton A., Iannuzzi L., King W.A. (2009). Classical and molecular cytogenetics of disorders of sex development in domestic animals. Cytogenet. Genome Res..

[13] Szczerbal I., Switonski M. (2016). Chromosome Abnormalities in Domestic Animals as Causes of Disorders of Sex Development or Impaired Fertility. Insights Anim. Reprod..

[14] Raudsepp T., Chowdhary B.P. (2016). Chromosome Aberrations and Fertility Disorders in Domestic Animals. Annu. Rev. Anim. Biosci..

[15] Raudsepp T., Finno C.J., Bellone R.R., Petersen J.L. (2019). Ten years of the horse reference genome: Insights into equine biology, domestication and population dynamics in the post-genome era. Anim. Genet..

[16] Kirillow S. (1912). Die Spermatogenese beim Pferde. Arch. Fuer Mikrosk. Anat..

[17] Masui K. (1919). A spermatogenesis of domestic mammals. I. The spermatogenesis of the horse (*Equus caballus*). J. Coll. Agric. Tokyo Imp. Univ..

[18] Painter T.S. (1924). Studies in mammalian spermatogenesis. V. The chromosomes of the horse. J. Exp. Zool..

[19] Wodsedalek J.E. (1914). Spermatogenesis of the horse with special reference to the accessory chromosome and the chromatoid body. Biol. Bull..

[20] Makino S. (1942). The chromosomes of the horse (*Equus caballus*). Cytologia.

[21] Rothfels K.H., Alexrad A.A., Siminovitch L., Parker R.C.M. (1959). The origin of altered cell lines from mouse, monkey and man, as indicated by chromosome and transplantation studies. Proc. Can. Cancer Res. Conf..

[22] Moorhead P.S., Nowell P.C., Mellman W.J., Battips D.M., Hungerford D.A. (1960). Chromosome preparations of leucocytes cultured from human peripheral blood. Exp. Cell Res..

[23] Makino S., Sofuni T., Sasaki M.S. (1963). A revised study of the chromosomes of the horse, the ass and the mule. Proc. Jpn. Acad..

[24] Seabright M. (1971). A rapid banding technique for human chromosomes. Lancet.

[25] Schweizer D., Ambros P., Andrle M. (1978). Modification of DAPI banding on human chromosomes by prestaining with a DNA-binding oligopeptide antibiotic, distamycin A. Exp. Cell Res..

[26] Arrighi F.E., Hsu T.C. (1971). Localization of heterochromatin in human chromosomes. Cytogenetics.

[27] Mendoza M.N., Schalnus S.A., Thomson B., Bellone R.R., Juras R., Raudsepp T. (2020). Novel complex unbalanced dicentric X-autosome rearrangement in a Thoroughbred mare with a mild effect on the phenotype. Cytogenet. Genome Res..

[28] Raudsepp T., Durkin K., Lear T.L., Das P.J., Avila F., Kachroo P., Chowdhary B.P. (2010). Molecular heterogeneity of XY sex reversal in horses. Anim. Genet..

[29] Dutrillaux B., Lejeune J. (1971). A new technic of analysis of the human karyotype. C R Acad. Hebd. Seances Acad. Sci. D.

[30] Goodpasture C., Bloom S.E. (1975). Visualization of nucleolar organizer regions im mammalian chromosomes using silver staining. Chromosoma.

[31] Iannuzzi L., di Meo G.P., Perucatti A., Incarnato D., Peretti V., Ciotola F., Barbieri V. (2003). An improved characterization of horse (*Equus caballus*, 2n = 64) chromosomes by using replicating G and R banding patterns. Caryologia.

[32] Rønne M. (1992). Putative fragile sites in the horse karyotype. Hereditas.

[33] Wnuk M., Villagomez D.A., Bugno-Poniewierska M., Tumidajewicz P., Carter T.F., Slota E. (2012). Nucleolar organizer regions (NORs) distribution and behavior in spermatozoa and meiotic cells of the horse (*Equus caballus*). Theriogenology.

[34] Ford C.E., Pollock D.L., Gustavsson I. (1980). Proceedings of the First International Conference for the Standardisation of Banded Karyotypes of Domestic Animals. University of Reading Reading, England, 2nd–6th August 1976. Hereditas.

[35] Richer C.L., Power M.M., Klunder L.R., McFeely R.A., Kent M.G. (1990). Standard karyotype of the domestic horse (*Equus caballus*). Committee for standardized karyotype of *Equus caballus*. The Second International Conference for Standardization of Domestic Animal Karyotypes, INRA, Jouy-en Josas, France, 22nd–26th May 1989. Hereditas.

[36] Bowling A.T., Breen M., Chowdhary B.P., Hirota K., Lear T., Millon L.V., Ponce D.L.F., Raudsepp T., Stranzinger G. (1997). International system for cytogenetic nomenclature of the domestic horse. Report of the Third International Committee for the Standardization of the domestic horse karyotype, Davis, CA, USA 1996. Chromosome Res..

[37] Das P.J., Lyle S.K., Beehan D., Chowdhary B.P., Raudsepp T. (2012). Cytogenetic and molecular characterization of Y isochromosome in a 63XO/64Xi(Yq) mosaic karyotype of an intersex horse. Sex. Dev..

[38] Janečka J.E., Davis B.W., Ghosh S., Paria N., Das P.J., Orlando L., Schubert M., Nielsen M.K., Stout T.A.E., Brashear W. (2018). Horse Y chromosome assembly displays unique evolutionary features and putative stallion fertility genes. Nat. Commun..

[39] Wade C.M., Giulotto E., Sigurdsson S., Zoli M., Gnerre S., Imsland F., Lear T.L., Adelson D.L., Bailey E., Bellone R.R. (2009). Genome sequence, comparative analysis, and population genetics of the domestic horse. Science.

[40] Deryusheva S.E., Loginova Y.A., Chiryaeva O.G., Yaschak K., Smirnov A.F. (1997). Distribution of ribosomal RNA genes on chromosomes of domestic horse (*Equus caballus*) revealed by fluorescence in situ hybridization. Genetika.

[41] Loginova J., Derjusheva S., Jasczak K. (1996). Some cases of NOR instability in horse chromosomes. Cytogenet. Cell Genet..

[42] Payne H.W., Ellsworth K., DeGroot A. (1968). Aneuploidy in an infertile mare. J. Am. Vet. Assoc..

[43] Chandley A.C., Fletcher J., Rossdale P.D., Peace C.K., Ricketts S.W., McEnery R.J., Thorne J.P., Short R.V., Allen W.R. (1975). Chromosome abnormalities as a cause of infertility in mares. J. Reprod. Fertil. Suppl..

[44] Bowling A.T., Millon L., Hughes J.P. (1987). An update of chromosomal abnormalities in mares. J. Reprod. Fertil. Suppl..

[45] Parada R., Jaszczak K., Sysa P., Jaszczak J. (1996). Cytogenetic investigations of mares with fertility disturbances. Pr. Mat. Zoot..

[46] Bugno M., Slota E., Koscielny M. (2007). Karyotype evaluation among young horse populations in Poland. Schweiz. Arch. Fur Tierheilkd..

[47] Wieczorek M., Switonski M., Yang F. (2001). A low-level X chromosome mosaicism in mares, detected by chromosome painting. J. Appl. Genet..

[48] Pawlak M., Rogalska-Niżnik N., Cholewiński G., Świtoński M. (2000). Study on the origin of 64,XX/63,X karyotype in four sterile mares. Vet. Med. Czech..

[49] Bugno-Poniewierska M., Wojtaszek M., Pawlina-Tyszko K., Kowalska K., Witarski W., Raudsepp T. Evaluation of the prevalence of sex chromosome aberrations in a population of young horses—Preliminary results. Proceedings of the Doroty Russell Havemeyer 12th International Horse Genome Workshop.

[50] Wojtaszek M., Kowalska K., Witarski W., Bugno-Poniewierska M. (2017). Diagnostics of horse karyotype aberrations—The initial results of screening. Wiadomości Zootech. R. Lv..

[51] Raudsepp T., Das P.J., Avila F., Chowdhary B.P. (2012). The pseudoautosomal region and sex chromosome aneuploidies in domestic species. Sex. Dev..

[52] Ruiz A.J., Castaneda C., Raudsepp T., Tibary A. (2019). Azoospermia and Y chromosome-autosome translocation in a Friesian stallion. J. Equine Vet. Sci..

[53] Juras R., Raudsepp T., Das P.J., Conant E., Cothran E.G. (2010). XX/XY Blood Lymphocyte Chimerism in Heterosexual Dizygotic Twins from an American Bashkir Curly Horse. Case Report. J. Equine Vet. Sci..

[54] Ghosh S., Carden C.F., Juras R., Mendoza M.N., Jevit M.J., Castaneda C., Phelps O., Dube J., Kelley D.E., Varner D.D. (2020). Two Novel Cases of Autosomal Translocations in the Horse: Warmblood Family Segregating t(4;30) and a Cloned Arabian with a de novo t(12;25). Cytogenet. Genome Res..

[55] Ghosh S., Das P.J., Avila F., Thwaits B.K., Chowdhary B.P., Raudsepp T. (2016). A Non-Reciprocal Autosomal Translocation 64,XX, t(4;10)(q21;p15) in an Arabian Mare with Repeated Early Embryonic Loss. Reprod. Domest. Anim..

[56] Lear T.L., Raudsepp T., Lundquist J.M., Brown S.E. (2014). Repeated early embryonic loss in a thoroughbred mare with a chromosomal translocation [64, XX, t (2; 13)]. J. Equine Vet. Sci..

[57] Brito L.F.C., Sertich P.L., Durkin K., Chowdhary B.P., Turner R.M., Greene L.M., McDonnell S. (2008). Autosomic 27 trisomy in a standardbred colt. J. Equine Vet. Sci..

[58] Ghosh S., Davis B.W., Rosengren M., Jevit M.J., Castaneda C., Arnold C., Jaxheimer J., Love C.C., Varner D.D., Lindgren G. (2020). Characterization of A Homozygous Deletion of Steroid Hormone Biosynthesis Genes in Horse Chromosome 29 as A Risk Factor for Disorders of Sex Development and Reproduction. Genes.

[59] Gill J.J., Kempski H.M., Hallows B.J., Warren A.M. (1988). A 64XX/65XXX mosaic mare (*Equus caballus*) and associated infertility. Equine Vet. J..

[60] Höhn H., Klug E., Rieck G.W. A 63,XO/65,XYY mosaic in a case of questionable equine male pseudohermaphroditism. Proceedings of the 4 th European Colloquium on Cytogenetics of Domestic Animals.

[61] Paget S., Ducos A., Mignotte F., Raymond I., Pinton A., Seguela A., Berland H.M., Brun-Baronnat C., Darre A., Darre R. (2001). 63,XO/65,XYY mosaicism in a case of equine male pseudohermaphroditism. Vet. Rec..

[62] Stewart-Scott I.A. (1988). Infertile mares with chromosome abnormalities. N. Z. Vet. J..

[63] Halnan C.R.E., Watson J.I., Pryde L.C. (1982). Detection by G-and C-band karyotyping of gonosome anomalies in horses of different breeds. J. Reprod. Fert. Suppl..

[64] Bugno M., Zabek T., Golonka P., Pienkowska-Schelling A., Schelling C., Slota E. (2008). A case of an intersex horse with 63,X/64,XX/65,XX,del(Y)(q?) karyotype. Cytogenet. Genome Res..

[65] Klunder L.R., McFeely R.A., Willard J.P. (1990). Six separate sex chromosome anomalies in an Arabian mare. Equine Vet. J..

[66] Neuhauser S., Handler J., Schelling C., Pienkowska-Schelling A. (2019). Fertility and 63,X Mosaicism in a Haflinger Sibship. J. Equine Vet. Sci.

[67] Mushtag A., Memon B.V. (1994). Sterility Associated with 64,XX/64,XY,63,X0 mosaic karyotype in a Belgian mare. Equine Pract..

[68] Mäkinen A., Hasegawa T., Syriä P., Katila T. (2006). Infertile mares with XO and XY sex chromosome deviations. Equine Vet. Educ..

[69] Jaszczak K., Sysa P. (1992). Anomalie chromosomowe u koni i ich skutki w reprodukcji. Przegląd. Hod..

[70] Halnan C.R. (1985). Equine cytogenetics: Role in equine veterinary practice. Equine Vet. J..

[71] Bugno M., Słota E., Ząbek T. (2001). Two cases of subfertile mares with 64,XX/63,X mosa-ic karyotype. Ann. Anim. Sci..

[72] Breen M., Langford C.F., Carter N.P., Fischer P.E., Marti E., Gerstenberg C., Allen W.R., Lear T.L., Binns M.M. (1997). Detection of equine X chromosome abnormalities in equids using a horse X whole chromosome paint probe (WCPP). Vet. J..

[73] Mäkinen A., Hasegawa T., Makila M., Katila T. (1999). Infertility in two mares with XY and XXX sex chromosomes. Equine Vet. J..

[74] Bugno M., Slota E., Wieczorek M., Yang F., Buczynski J., Switonski M. (2003). Nonmosaic X trisomy, detected by chromosome painting, in an infertile mare. Equine Vet. J..

[75] De Lorenzi L., Molteni L., Zannotti M., Galli C., Parma P. (2010). X trisomy in a sterile mare. Equine Vet. J..

[76] Kubien E.M., Pozor M.A., Tischner M. (1993). Clinical, cytogenetic and endocrine evaluation of a horse with a 65,XXY karyotype. Equine Vet. J..

[77] Mäkinen A., Katila T., Andersson M., Gustavsson I. (2000). Two sterile stallions with XXY-syndrome. Equine Vet. J..

[78] Iannuzzi L., Di Meo G.P., Perucatti A., Spadetta M., Incarnato D., Parma P., Iannuzzi A., Ciotola F., Peretti V., Perrotta G. (2004). Clinical, cytogenetic and molecular studies on sterile stallion and mare affected by XXY and sex reversal syndromes, respectively. Caryologia.

[79] Bouters R., Vandeplassche M., de Moor A. (1972). An intersex (male pseudohermaphrodite) horse with 64 XX-65 XXY mosaicism. Equine Vet. J..

[80] Bielański W., Kleczkowska A., Tischner M., Jagiarz M. (1980). Comparative cytogenetic examinations of parents and sibilding of colt with a false masculine hermaphroditism. Med. Wet..

[81] Bugno M., Pieńkowska-Schelling A., Schelling C., Włodarczyk N., Słota E. (2006). A probe generated by chromosome microdissection, useful for detection of equine X chromosome aneuploidy. Ann. Anim. Sci..

[82] Basrur P.K., Kanagawa H., Gilman J.P. (1969). An equine intersex with unilateral gonadal agenesis. Can. J. Comp. Med..

[83] Dunn H.O., Vaughan J.T., McEntee K. (1974). Bilaterally cryptorchid stallion with female karyotype. Cornell Vet..

[84] Klunder L.R., McFeeley R.A. (1989). Chromosome analysis of 130 equine clinical cases. Proceedings of the 6th North American Colloquium on Cytogenetics of Domestic Animals.

[85] Chandley A.C. (1984). Infertility and chromosome abnormality. Oxf. Rev. Rep. Biol..

[86] Nicolas F.W. (1987). Veterinary Genetics.

[87] Marx M.B., Melnyk J., Persinger G., Ono S., McGee W., Kaufman W., Pessin A., Gillespie R. (1973). Cytogenetics of the superhorse. J. Hered..

[88] Shilton C.A., Kahler A., Davis B.W., Crabtree J.R., Crowhurst J., McGladdery A.J., Wathes D.C., Raudsepp T., de Mestre A.M. (2020). Whole genome analysis reveals aneuploidies in early pregnancy loss in the horse. Sci. Rep..

[89] Colley E., Hamilton S., Smith P., Morgan N.V., Coomarasamy A., Allen S. (2019). Potential genetic causes of miscarriage in euploid pregnancies: A systematic review. Hum. Reprod. Update.

[90] Hyde K.J., Schust D.J. (2015). Genetic considerations in recurrent pregnancy loss. Cold Spring Harb. Perspect. Med..

[91] Power M.M. (1987). Equine half sibs with an unbalanced X;15 translocation or trisomy 28. Cytogenet. Cell Genet..

[92] Bowling A.T., Millon L.V. (1990). Two autosomal trisomies in the horse: 64,XX,-26,+t(26q26q) and 65,XX,+30. Genome.

[93] Zhang T.Q., Bellamy J., Buwn L.C., Weber A.F., Ruth G.R. Autosomal trisomy in a foal with contracted tendon syndrome. Proceedings of the 10th European Colloqium on Cytogenetics of Domestic Animals.

[94] Buoen L.C., Zhang T.Q., Weber A.F., Turner T., Bellamy J., Ruth G.R. (1997). Arthrogryposis in the foal and its possible relation to autosomal trisomy. Equine Vet. J..

[95] Holl H.M., Lear T.L., Nolen-Walston R.D., Slack J., Brooks S.A. (2013). Detection of two equine trisomies using SNP-CGH. Mamm. Genome.

[96] Lear T.L., Cox J.H., Kennedy G.A. (1999). Autosomal trisomy in a Thoroughbred colt: 65,XY,+31. Equine Vet. J..

[97] Kubien E.M., Tischner M. (2002). Reproductive success of a mare with a mosaic karyotype: 64,XX/65,XX,+30. Equine Vet. J..

[98] Cuckle H., Morris J. (2020). Maternal age in the epidemiology of common autosomal trisomies. Prenat. Diagn..

[99] Weckselblatt B., Rudd M.K. (2015). Human Structural Variation: Mechanisms of Chromosome Rearrangements. Trends Genet..

[100] Morin S.J., Eccles J., Iturriaga A., Zimmerman R.S. (2017). Translocations, inversions and other chromosome rearrangements. Fertil. Steril..

[101] King W.A. (2008). Chromosome variation in the embryos of domestic animals. Cytogenet. Genome Res..

[102] Power M.M. (1991). The first description of a balanced reciprocal translocation [t(1q;3q)] and its clinical effects in a mare. Equine Vet. J..

[103] Long S.E. (1996). Tandem 1;30 translocation: A new structural abnormality in the horse (*Equus caballus*). Cytogenet. Cell Genet..

[104] Lear T.L., Layton G. (2002). Use of zoo-FISH to characterise a reciprocal translocation in a thoroughbred mare: T(1;1 6)(q16;q21.3). Equine Vet. J..

[105] Lear T.L., Lundquist J., Zent W.W., Fishback W.D., Clark A. (2008). Three autosomal chromosome translocations associated with repeated early embryonic loss (REEL) in the domestic horse (*Equus caballus*). Cytogenet. Genome Res..

[106] Lyon M.F. (1961). Gene action in the X-chromosome of the mouse (*Mus musculus* L.). Nature.

[107] Lyon M.F. (1962). Sex chromatin and gene action in the mammalian X-chromosome. Am. J. Hum. Genet..

[108] Cantone I., Fisher A.G. (2017). Human X chromosome inactivation and reactivation: Implications for cell reprogramming and disease. Philos. Trans. R. Soc. Lond. B Biol. Sci..

[109] Carrel L., Brown C.J. (2017). When the Lyon(ized chromosome) roars: Ongoing expression from an inactive X chromosome. Philos Trans. R. Soc. Lond. B Biol. Sci..

[110] Waters P.D., Ruiz-Herrera A. (2020). Meiotic Executioner Genes Protect the Y from Extinction. Trends Genet..

[111] Donaldson B., Villagomez D.A.F., Revay T., Rezaei S., King W.A. (2019). Non-Random Distribution of Reciprocal Translocation Breakpoints in the Pig Genome. Genes.

[112] Ghosh S., Qu Z., Das P.J., Fang E., Juras R., Cothran E.G., McDonell S., Kenney D.G., Lear T.L., Adelson D.L. (2014). Copy number variation in the horse genome. PLoS Genet..

[113] McFeely R.A., Klunder L.R., Byars D. (1989). Equine infertility associated with autosomal mixoploidy. Proceedings of the 6th North American Colloquium on Domestic Animal Cytogenetics.

[114] Brooks S.A., Lear T.L., Adelson D.L., Bailey E. (2007). A chromosome inversion near the KIT gene and the Tobiano spotting pattern in horses. Cytogenet. Genome Res..

[115] McGowan-Jordan J., Hastings R.J., Moore S., ISCN (2020). An International System for Human Cytogenomic Nomenclature.

[116] Mäkela O., Gustavsson I., Hollmen T. (1994). A 64,X,i(Xq) karyotype in a standardbred filly. Equine Vet. J..

[117] Herzog A., Hohn H., Klug E., Hecht W. (1989). A sex chromosome mosaic in male pseudohermaphroditism in a horse. Tierarztl. Prax..

[118] Bugno-Poniewierska M., Ząbek T., Pawlina K., Klukowska-Rötzler J., Rojek M., Słota E. Y isochromosome in mare with sex chromosome mosaicism 63,X/64,XYqi. Proceedings of the 19th International Colloquium on Animal Cytogenetics and Gene Mapping.

[119] Riggs P.K., Rønne M. (2009). Fragile sites in domestic animal chromosomes: Molecular insights and challenges. Cytogenet. Genome Res..

[120] Durkin S.G., Glover T.W. (2007). Chromosome fragile sites. Annu. Rev. Genet..

[121] Santagostino M., Piras F.M., Cappelletti E., del Giudice S., Semino O., Nergadze S.G., Giulotto E. (2020). Insertion of Telomeric Repeats in the Human and Horse Genomes: An Evolutionary Perspective. Int. J. Mol. Sci..

[122] Malan V., Vekemans M., Turleau C. (2006). Chimera and other fertilization errors. Clin. Genet..

[123] Keszka J., Jaszczak K., Klewiec J. (2001). High frequency of lymphocyte chimerism XX/XY and an analysis of hereditary occurrence of placental anastomoses in Booroola sheep. J. Anim. Breed. Genet. Z. Fur Tierz. Und. Zucht..

[124] Komisarek J., Dorynek Z. (2002). Genetic aspects of twinning in cattle. J. Appl. Genet..

[125] Albarella S., de Lorenzi L., Catone G., Magi G.E., Petrucci L., Vullo C., D’Anza E., Parma P., Raudsepp T., Ciotola F. (2018). Diagnosis of XX/XY Blood Cell Chimerism at a Low Percentage in Horses. J. Equine Vet. Sci..

[126] Demyda-Peyras S., Anaya G., Bugno-Poniewierska M., Pawlina K., Membrillo A., Valera M., Moreno-Millan M. (2014). The use of a novel combination of diagnostic molecular and cytogenetic approaches in horses with sexual karyotype abnormalities: A rare case with an abnormal cellular chimerism. Theriogenology.

[127] Demyda-Peyras S., Membrillo A., Bugno-Poniewierska M., Pawlina K., Anaya G., Moreno-Millan M. (2013). The use of molecular and cytogenetic methods as a valuable tool in the detection of chromosomal abnormalities in horses: A case of sex chromosome chimerism in a Spanish purebred colt. Cytogenet. Genome Res..

[128] Bowling A.T., Stott M.L., Bickel L. (1993). Silent blood chimaerism in a mare confirmed by DNA marker analysis of hair bulbs. Anim. Genet..

[129] Bugno M., Slota E., Tischner M., Kozubska-Sobocinska A. (1999). A case of 64,XX/64,XY leucocytic chimerism in a fertile mare of the Wielkopolska breed. Ann. Anim. Sci..

[130] Gustavsson I. (1977). Fertility of sires born as dizygotic twins and sex ratio in their progeny groups. Ann. Genet. Sel Anim.

[131] Zhang T., Buoen L.C., Seguin B.E., Ruth G.R., Weber A.F. (1994). Diagnosis of freemartinism in cattle: The need for clinical and cytogenic evaluation. J. Am. Vet. Med. Assoc..

[132] Lee P.A., Houk C.P., Ahmed S.F., Hughes I.A. (2006). International Consensus Conference on Intersex organized by the Lawson Wilkins Pediatric Endocrine, and E. the European Society for Paediatric. Consensus statement on management of intersex disorders. International Consensus Conference on Intersex. Pediatrics.

[133] Villagomez D.A., Lear T.L., Chenier T., Lee S., McGee R.B., Cahill J., Foster R.A., Reyes E., John E.S., King W.A. (2011). Equine disorders of sexual development in 17 mares including XX, SRY-negative, XY, SRY-negative and XY, SRY-positive genotypes. Sex. Dev..

[134] Raudsepp T. (2020). Genetics of Equine Reproductive Diseases. Vet. Clin. N. Am. Equine Pract..

[135] Power M.M. (1986). XY sex reversal in a mare. Equine Vet. J..

[136] Kent M.G., Shoffner R.N., Buoen L., Weber A.F. (1986). XY sex-reversal syndrome in the domestic horse. Cytogenet. Cell Genet..

[137] Kent M.G., Shoffner R.N., Hunter A., Elliston K.O., Schroder W., Tolley E., Wachtel S.S. (1988). XY sex reversal syndrome in the mare: Clinical and behavioral studies, H-Y phenotype. Hum. Genet..

[138] Pailhoux E., Cribiu E.P., Parma P., Cotinot C. (1995). Molecular analysis of an XY mare with gonadal dysgenesis. Hereditas.

[139] Meyers-Wallen V.N., Hurtgen J., Schlafer D., Tulleners E., Cleland W.R., Ruth G.R., Acland G.M. (1997). Sry-negative XX true hermaphroditism in a Pasa Fino horse. Equine Vet. J..

[140] Abe S., Miyake Y.I., Kageyama S.I., Watanabe G., Taya K., Kawakura K. (1999). Deletion of the Sry region on the Y chromosome detected in a case of equine gonadal hypoplasia (XY female) with abnormal hormonal profiles. Equine Vet. J..

[141] Anaya G., Moreno-Millan M., Bugno-Poniewierska M., Pawlina K., Membrillo A., Molina A., Demyda-Peyras S. (2014). Sex reversal syndrome in the horse: Four new cases of feminization in individuals carrying a 64,XY SRY negative chromosomal complement. Anim. Reprod. Sci..

[142] Pienkowska-Schelling A., Becker D., Bracher V., Pineroli B., Schelling C. (2014). Cytogenetical and molecular analyses in a horse with SRY-negative sex reversal. Schweiz. Arch. Tierheilkd..

[143] Martinez M.M., Costa M., Ratti C. (2020). Molecular screening of XY SRY-negative sex reversal cases in horses revealed anomalies in amelogenin testing. J. Vet. Diagn. Investig..

[144] Bugno M., Klukowska J., Slota E., Tischner M., Switonski M. (2003). A sporadic case of the sex-reversed mare (64,XY.; SRY-negative): Molecular and cytogenetic studies of the Y chromosome. Theriogenology.

[145] Sant’Anna Monteiro da Silva E., Delfiol D.J.Z., Fabris V.H., Santos B.M., Nogueira G.M., Guimaraes G.B.O., Nogueira P.P.d., da Mota L.S.L.S. (2020). Teratoma Associated With Testicular Tissue in a Female-Like Horse With 64,XY (SRY-Positive) Disorder of Sex Development. J. Equine Vet. Sci..

[146] Knobbe M.G., Maenhoudt C., Turner R.M., McDonnell S.M. (2011). Physical, behavioral, endocrinologic, and cytogenetic evaluation of two Standardbred racehorses competing as mares with an intersex condition and high postrace serum testosterone concentrations. J. Am. Vet. Med. Assoc..

[147] Howden K.J. (2004). Androgen insensitivity syndrome in a thoroughbred mare (64, XY--testicular feminization). Can. Vet. J..

[148] Crabbe B.G., Freeman D.A., Grant B.D., Kennedy P., Whitlatch L., MacRae K. (1992). Testicular feminization syndrome in a mare. J. Am. Vet. Med. Assoc..

[149] Switonski M., Chmurzynska A., Szczerbal I., Lipczynski A., Yang F., Nowicka-Posluszna A. (2005). Sex reversal syndrome (64,XY.; SRY-positive) in a mare demonstrating masculine behaviour. J. Anim. Breed. Genet..

[150] Révay T., Villagomez D.A., Brewer D., Chenier T., King W.A. (2012). GTG mutation in the start codon of the androgen receptor gene in a family of horses with 64,XY disorder of sex development. Sex. Dev..

[151] Bolzon C., Joone C.J., Schulman M.L., Harper C.K., Villagomez D.A., King W.A., Revay T. (2016). Missense Mutation in the Ligand-Binding Domain of the Horse Androgen Receptor Gene in a Thoroughbred Family with Inherited 64,XY (SRY+) Disorder of Sex Development. Sex. Dev..

[152] Welsford G.E., Munk R., Villagomez D.A., Hyttel P., King W.A., Revay T. (2017). Androgen Insensitivity Syndrome in a Family of Warmblood Horses Caused by a 25-bp Deletion of the DNA-Binding Domain of the Androgen Receptor Gene. Sex. Dev..

[153] Terribile M., Stizzo M., Manfredi C., Quattrone C., Bottone F., Giordano D.R., Bellastella G., Arcaniolo D., de Sio M. (2019). 46,XX Testicular Disorder of Sex Development (DSD): A Case Report and Systematic Review. Medicina (Kaunas).

[154] Bannasch D., Rinaldo C., Millon L., Latson K., Spangler T., Hubberty S., Galuppo L., Lowenstine L. (2007). SRY negative 64,XX intersex phenotype in an American saddlebred horse. Vet. J..

[155] Buoen L.C., Zhang T.Q., Weber A.F., Ruth G.R. (2000). SRY-negative, XX intersex horses: The need for pedigree studies to examine the mode of inheritance of the condition. Equine Vet. J..

[156] Jaszczak K., Sysa P., Sacharczuk M., Parada R., Romanowicz K., Kawka M., Jarmuz W. (2010). SRY-negative, 64,XX sex reversal in a Konik Polski horse: A case report. Anim. Sci. Pap. Rep..

[157] Vaughan L., Schofield W., Ennis S. (2001). SRY-negative XX sex reversal in a pony: A case report. Theriogenology.

[158] Peretti V., Satue K., Ciotola F., Cristarella S., de Majo M., Biondi V., D’Anza E., Albarella S., Quartuccio M. (2020). An Unusual Case of Testicular Disorder in Sex Development of Arabian Mare (64,XX SRY-Negative). Animals.

[159] Ciotola F., Albarella S., Pasolini M.P., Auletta L., Esposito L., Iannuzzi L., Peretti V. (2012). Molecular and cytogenetic studies in a case of XX SRY-negative sex reversal in an Arabian horse. Sex. Dev..

[160] Torres A., Silva J.F., Bernardes N., Luis J.S., da Costa L.L. (2013). 64, XX, SRY-negative, testicular DSD syndrome in a Lusitano horse. Reprod. Domest. Anim..

[161] Huber D., von Voithenberg L.V., Kaigala G.V. (2018). Fluorescence in situ hybridization (FISH): History, limitations and what to expect from micro-scale FISH?. Micro Nano Eng..

[162] Raudsepp T., Chowdhary B.P. (2008). FISH for mapping single copy genes. Methods Mol. Biol..

[163] Rubes J., Pinton A., Bonnet-Garnier A., Fillon V., Musilova P., Michalova K., Kubickova S., Ducos A., Yerle M. (2009). Fluorescence in situ hybridization applied to domestic animal cytogenetics. Cytogenet. Genome Res..

[164] Raudsepp T., Gustafson-Seabury A., Durkin K., Wagner M.L., Goh G., Seabury C.M., Brinkmeyer-Langford C., Lee E.J., Agarwala R., Stallknecht-Rice E. (2008). A 4,103 marker integrated physical and comparative map of the horse genome. Cytogenet. Genome Res..

[165] Leeb T., Vogl C., Zhu B., de Jong P.J., Binns M.M., Chowdhary B.P., Scharfe M., Jarek M., Nordsiek G., Schrader F. (2006). A human-horse comparative map based on equine BAC end sequences. Genomics.

[166] Yang F., Fu B., O’Brien P.C., Nie W., Ryder O.A., Ferguson-Smith M.A. (2004). Refined genome-wide comparative map of the domestic horse, donkey and human based on cross-species chromosome painting: Insight into the occasional fertility of mules. Chromosome Res..

[167] Bugno M., Slota E., Pienkowska-Schelling A., Schelling C. (2009). Identification of chromosome abnormalities in the horse using a panel of chromosome-specific painting probes generated by microdissection. Acta Vet. Hung..

[168] Raudsepp T., Chowdhary B.P. (1999). Construction of chromosome-specific paints for meta- and submetacentric autosomes and the sex chromosomes in the horse and their use to detect homologous chromosomal segments in the donkey. Chromosome Res..

[169] Bugno M., Slota E., Pienkowska-Schelling A., Schelling C. (2007). Detection of equine X chromosome mosaicism in a mare using an equine X whole chromosome painting probe (WCPP)—A case report. Acta Vet. Hung..

[170] Pienkowska-Schelling A., Bugno M., Owczarek-Lipska M., Schelling C., Slota E. (2006). Probe generated by Y chromosome microdissection is useful for analysing the sex chromosomes of the domestic horse. J. Anim. Feed Sci..

[171] Bugno M., Slota E. (2007). Application of arm-specific painting probes of horse X chromosome for karyotype analysis in an infertile Hutsul mare with 64,XX/65,XX+Xp karyotype: Case report. Acta Vet. Hung..

[172] Alkan C., Cardone M.F., Catacchio C.R., Antonacci F., O’Brien S.J., Ryder O.A., Purgato S., Zoli M., della Valle G., Eichler E.E. (2011). Genome-wide characterization of centromeric satellites from multiple mammalian genomes. Genome Res..

[173] Bugno-Poniewierska M., Wnuk M., Witarski W., Slota E. (2009). The fluorescence in situ study of highly repeated DNA sequences in domestic horse (*Equus caballus*) and domestic donkey (*Equus asinus*)—Advantages and limits of usefulness in phylogenetic analyses. J. Anim. Feed Sci..

[174] Wnuk M., Bugno M., Slota E. (2008). Application of primed in situ DNA synthesis (PRINS) with telomere human commercial kit in molecular cytogenetics of Equus caballus and Sus scrofa scrofa. Folia Histochem. Cytobiol..

[175] Bugno-Poniewierska M., Zabek T., Semik E., Pawlina K., Tischner M. (2014). A case of sex chromosome mosaicism 64,XX/65,XXY/66,XXYY in mare. Chromosome Res..

[176] Witarski W., Kij B., Nowak A., Bugno-Poniewierska M. (2020). Premature centromere division (PCD) identified in a hucul mare with reproductive difficulties. Reprod. Domest. Anim..

[177] Wyrobek A.J., Alhborn T., Balhorn R., Stanker L., Pinkel D. (1990). Fluorescence in situ hybridization to Y chromosomes in decondensed human sperm nuclei. Mol. Reprod. Dev..

[178] Bugno-Poniewierska M., Jablonska Z., Slota E. (2009). Modification of equine sperm chromatin decondensation method to use fluorescence in situ hybridization (FISH). Folia Histochem. Cytobiol..

[179] Bugno M., Jablonska Z., Tischner M., Klukowska-Rotzler J., Pienkowska-Schelling A., Schelling C., Slota E. (2010). Detection of sex chromosome aneuploidy in equine spermatozoa using fluorescence in situ hybridization. Reprod. Domest. Anim..

[180] Bugno-Poniewierska M., Kozub D., Pawlina K., Tischner M., Tischner M., Slota E., Wnuk M. (2011). Determination of the correlation between stallion’s age and number of sex chromosome aberrations in spermatozoa. Reprod. Domest. Anim..

[181] Bugno-Poniewierska M., Pawlina K., Tischner M., Tischner M. (2014). Age-related effects on sex chromosome aberrations in equine spermatozoa. J. Equine Vet. Sci..

[182] Kjöllerström H.J., Oom M.d.M., Chowdhary B.P., Raudsepp T. (2016). Fertility Assessment in Sorraia Stallions by Sperm-Fish and Fkbp6 Genotyping. Reprod. Domest. Anim..

[183] Kallioniemi A., Kallioniemi O.P., Sudar D., Rutovitz D., Gray J.W., Waldman F., Pinkel D. (1992). Comparative genomic hybridization for molecular cytogenetic analysis of solid tumors. Science.

[184] Bugno-Poniewierska M., Staron B., Potocki L., Gurgul A., Wnuk M. (2016). Identification of Unbalanced Aberrations in the Genome of Equine Sarcoid Cells Using Cgh Technique. Ann. Anim. Sci..

[185] Pinkel D., Albertson D.G. (2005). Comparative genomic hybridization. Annu. Rev. Genom. Hum. Genet..

[186] Pinkel D., Albertson D.G. (2005). Array comparative genomic hybridization and its applications in cancer. Nat. Genet..

[187] Bugno-Poniewierska M., Ząbek T., Gurgul A., Pawlina K. Characteristics of chromosome rearrangements of an intersex horse using molecular cytogenetic techniques. Proceedings of the 22nd International Colloquium on Animal Cytogenetics and Genomics.

[188] Schaefer R.J., Schubert M., Bailey E., Bannasch D.L., Barrey E., Bar-Gal G.K., Brem G., Brooks S.A., Distl O., Fries R. (2017). Developing a 670k genotyping array to tag ~2M SNPs across 24 horse breeds. BMC Genom..

[189] Villagomez D.A., Pinton A. (2008). Chromosomal abnormalities, meiotic behavior and fertility in domestic animals. Cytogenet. Genome Res..

[190] Purgato S., Belloni E., Piras F.M., Zoli M., Badiale C., Cerutti F., Mazzagatti A., Perini G., della Valle G., Nergadze S.G. (2015). Centromere sliding on a mammalian chromosome. Chromosoma.

[191] Giulotto E., Raimondi E., Sullivan K.F. (2017). The Unique DNA Sequences Underlying Equine Centromeres. Cent. Kinetochores.

[192] Al-Jaru A., Goodwin W., Skidmore J., Khazanehdari K. (2014). Distribution of MLH1 foci in horse male synaptonemal complex. Cytogenet. Genome Res..

[193] Al-Jaru A., Goodwin W., Skidmore J., Raudsepp T., Khazanehdari K. (2014). Male horse meiosis: Metaphase I chromosome configuration and chiasmata distribution. Cytogenet. Genome Res..

[194] Kakoi H., Hirota K., Gawahara H., Kurosawa M., Kuwajima M. (2005). Genetic diagnosis of sex chromosome aberrations in horses based on parentage test by microsatellite DNA and analysis of X- and Y-linked markers. Equine Vet. J..

[195] Anaya G., Molina A., Valera M., Moreno-Millan M., Azor P., Peral-Garcia P., Demyda-Peyras S. (2017). Sex chromosomal abnormalities associated with equine infertility: Validation of a simple molecular screening tool in the Purebred Spanish Horse. Anim. Genet..

[196] Gamo S., Tozaki T., Kakoi H., Hirota K.I., Nakamura K., Nishii N., Alumunia J., Takasu M. (2019). X monosomy in the endangered Kiso horse breed detected by a parentage test using sex chromosome linked genes and microsatellites. J. Vet. Med. Sci..

[197] Bowling A.T., Millon L.V., Dileanis S. (1997). Physical mapping of genetic markers to chromosome 30 using a trisomic horse and evidence for maternal origin of the extra chromosome. Chromosome Res..

[198] Raudsepp T., Chowdhary B.P. (2015). The Eutherian Pseudoautosomal Region. Cytogenet. Genome Res..

[199] Chowdhary B.P., Raudsepp T. (2008). The horse genome derby: Racing from map to whole genome sequence. Chromosome Res..

[200] Blackmon H., Brandvain Y. (2017). Long-Term Fragility of Y Chromosomes Is Dominated by Short-Term Resolution of Sexual Antagonism. Genetics.

[201] Bondy C.A., Cheng C. (2009). Monosomy for the X chromosome. Chromosome Res..

[202] Lupski J.R., Stankiewicz P. (2005). Genomic disorders: Molecular mechanisms for rearrangements and conveyed phenotypes. PLoS Genet..

[203] Bonomi M., Rochira V., Pasquali D., Balercia G., Jannini E.A., Ferlin A., Italia N.G.K. (2017). Klinefelter syndrome (KS): Genetics, clinical phenotype and hypogonadism. J. Endocrinol. Investig..

[204] Skuse D., Printzlau F., Wolstencroft J. (2018). Sex chromosome aneuploidies. Handb. Clin. Neurol..

[205] Lange J., Skaletsky H., van Daalen S.K., Embry S.L., Korver C.M., Brown L.G., Oates R.D., Silber S., Repping S., Page D.C. (2009). Isodicentric Y chromosomes and sex disorders as byproducts of homologous recombination that maintains palindromes. Cell.

[206] Michala L., Goswami D., Creighton S.M., Conway G.S. (2008). Swyer syndrome: Presentation and outcomes. BJOG.

[207] Mantere T., Neveling K., Pebrel-Richard C., Benoist M., van der Zande G., Kater-Baats E., Baatout I., van Beek R., Yammine T., Oorsprong M. (2020). Next generation cytogenetics: Genome-imaging enables comprehensive structural variant detection for 100 constitutional chromosomal aberrations in 85 samples. bioRxiv.

